# Neutrophil-intrinsic TNF receptor signaling orchestrates host defense against *Staphylococcus aureus*

**DOI:** 10.1126/sciadv.adf8748

**Published:** 2023-06-16

**Authors:** Christine Youn, Cristina Pontaza, Yu Wang, Dustin A. Dikeman, Daniel P. Joyce, Martin P. Alphonse, Meng-Jen Wu, Sabrina J. Nolan, Mohamed A. Anany, Michael Ahmadi, Jeremy Young, Aron Tocaj, Luis A. Garza, Harald Wajant, Lloyd S. Miller, Nathan K. Archer

**Affiliations:** ^1^ Department of Dermatology, Johns Hopkins University School of Medicine, 1550 Orleans Street, Baltimore, MD 21287, USA.; ^2^ Division of Molecular Internal Medicine, Department of Internal Medicine II, University Hospital Würzburg, Würzburg 97080, Germany.; ^3^ Department of Microbial Biotechnology, Institute of Biotechnology, National Research Center, El Buhouth Street, Dokki, 12622 Giza, Egypt.

## Abstract

*Staphylococcus aureus* is the leading cause of skin and soft tissue infections and is a major health burden due to the emergence of antibiotic-resistant strains. To address the unmet need of alternative treatments to antibiotics, a better understanding of the protective immune mechanisms against *S. aureus* skin infection is warranted. Here, we report that tumor necrosis factor (TNF) promoted protection against *S. aureus* in the skin, which was mediated by bone marrow–derived immune cells. Furthermore, neutrophil-intrinsic TNF receptor (TNFR) signaling directed immunity against *S. aureus* skin infections. Mechanistically, TNFR1 promoted neutrophil recruitment to the skin, whereas TNFR2 prevented systemic bacterial dissemination and directed neutrophil antimicrobial functions. Treatment with a TNFR2 agonist showed therapeutic efficacy against *S. aureus* and *Pseudomonas aeruginosa* skin infections, which involved increased neutrophil extracellular trap formation. Our findings revealed nonredundant roles for TNFR1 and TNFR2 in neutrophils for immunity against *S. aureus* and can be therapeutically targeted for protection against bacterial skin infections.

## Teaser

TNF receptors evoke divergent neutrophil immunity against bacterial infections.

## INTRODUCTION

*Staphylococcus aureus* (*S. aureus*) is the leading cause of skin and soft tissue infections (SSTI) as well as invasive infections causing endocarditis, osteomyelitis, and sepsis (*1*). The emergence of antibiotic-resistant *S. aureus* is a serious health burden, as methicillin-resistant *S. aureus* (MRSA) constitutes most of the clinical isolates in the United States and is a predominant cause of death due to antibiotic-resistant bacteria worldwide (*2*). Furthermore, SSTIs caused by MRSA account for more than 14 million outpatient and emergency room visits and greater than 750,000 hospital admissions in the United States annually (*3*, *4*). With the emergence of antibiotic-resistant *S. aureus* outpacing the development of novel antibiotics, there is an urgent need to better understand the protective immune mechanisms against *S. aureus* infections for the development of host-directed therapeutics as alternatives to antibiotics.

Tumor necrosis factor (TNF) is a pleiotropic cytokine that signals through its two cognate receptors, TNF receptor 1 (TNFR1) and TNF receptor 2 (TNFR2) (*5*, *6*). TNFR1 is constitutively expressed by the majority of nucleated cells, whereas TNFR2 expression is induced upon activation and restricted to specific immune cells [e.g., regulatory T cells (T_regs_), B cells, and macrophages] and nonimmune cells (e.g., endothelial cells, epithelial cells, and fibroblasts) (*7*–*11*). TNF is involved in the pathogenesis of autoimmune and inflammatory diseases such as rheumatoid arthritis, irritable bowel syndrome, psoriasis, psoriatic arthritis, and ankylosing spondylitis (*7*), which has led to the development of Food and Drug Administration–approved TNF inhibitors to treat these disorders (*12*). Notably, TNF inhibitor–treated patients have an increased risk of *S. aureus* skin colonization and infection (*13*, *14*), suggesting the involvement of TNF in protection against *S. aureus* in the skin. However, the TNF/TNFR-mediated mechanisms of host defense against *S. aureus* skin infections are not entirely understood.

Therefore, we sought to dissect the role of TNF and the differential contributions of TNFR1 and TNFR2 in protection against *S. aureus* skin by comparing mice deficient in TNF, TNFR1, and TNFR2 in a *S. aureus* intradermal infection mouse model (*15*, *16*). Furthermore, we evaluated the cell types that produced and responded to TNF in the skin during the *S. aureus* infection. Last, we determined the therapeutic potential of a TNFR2 agonist against bacterial skin infections and whether it affected host immune responses.

## RESULTS

### TNF promotes host defense against *S. aureus* skin infections

**Fig. 1.**
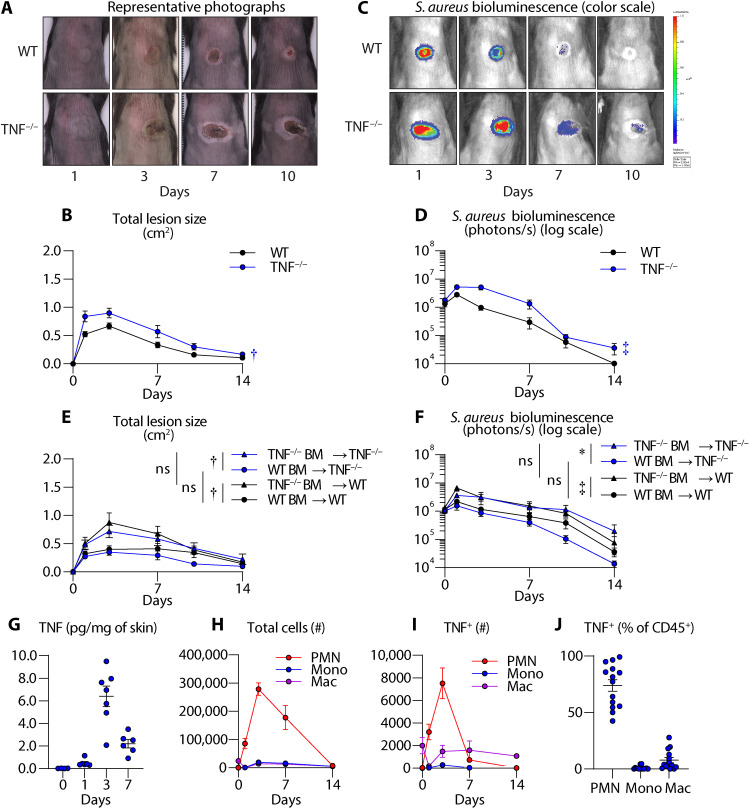
TNF from BM-derived immune cells is required for host defense against *S. aureus* skin infection. *S. aureus* (3 × 10^7^ CFU) skin infection was performed on WT and TNF-deficient (TNF^−/−^) mice (*n* ≥ 8 per group). (**A**) Representative photographs of skin lesions. (**B**) Mean total lesion size (cm^2^) ± SEM. (**C**) Representative in vivo BLI (photons/s) (log_10_ scale). (**D**) Mean total flux (photons/s) ± SEM. (**E** and **F**) BM-reconstituted WT or TNF^−/−^ recipient mice were intradermally inoculated with *S. aureus* (*n* ≥ 8). (E) Mean total lesion size (cm^2^) ± SEM. (F) Mean total flux (photons/s) ± SEM. (**G**) Mean TNF protein levels (picograms/milligram of tissue) ± SEM from WT *S. aureus*–infected skin. (**H** to **J**) Infected skin tissue from WT mice was harvested on days 0 through 14 for flow cytometry analysis (*n* ≥ 8 per group). (H) Total number of neutrophils (PMN), monocytes (Mono), and macrophages (Mac) ± SEM. (I) Total number of TNF^+^ neutrophils, monocytes, and macrophages ± SEM. (J) Mean TNF^+^ cells from the CD45^+^ population (%) ± SEM on day 3. *P < 0.05, †*P* < 0.01, ‡*P* < 0.001, as calculated by a two-way analysis of variance (ANOVA) test (B), (D), (E), and (F). Results are combined from at least two independent experiments. ns, not significant.

To determine whether TNF mediates protection against *S. aureus* skin infections, an intradermal *S. aureus* skin infection model was performed in wild-type (WT) and TNF-deficient (TNF^−/−^) mice (*15*, *16*). In this model, a bioluminescent community-acquired MRSA (CA-MRSA) USA300 LAC::*lux* strain is inoculated intradermally into the dorsal skin of mice. The bacterial burden is monitored noninvasively and longitudinally by in vivo bioluminescence imaging (BLI), which closely approximates ex vivo colony-forming unit (CFU) isolated from the infected skin [*R*^2^ = 0.9996] (*17*). We found that TNF^−/−^ mice developed significantly larger skin lesions and higher bacterial burdens than WT mice (Fig. 1, A to D), suggesting that TNF contributed to host defense against the *S. aureus* skin infection.

### Bone marrow cell–derived TNF is critical for host defense against *S. aureus* skin infections

TNF is expressed by immune cells, including neutrophils, monocytes, macrophages, and T cells, as well as nonimmune cells such as keratinocytes and endothelial cells (*18*–*21*). To determine the differential contribution of immune and nonimmune cells to TNF-mediated host defense, we used a bone marrow (BM) reconstitution experiment in which lethally irradiated WT and TNF^−/−^ mice were reconstituted with BM cells from WT or TNF^−/−^ mice to generate four groups: (i) TNF^−/−^ mice reconstituted with TNF^−/−^ BM (TNF^−/−^ BM → TNF^−/−^), (ii) TNF^−/−^ mice reconstituted with WT BM (WT BM → TNF^−/−^), (iii) WT mice reconstituted with TNF^−/−^ BM (TNF^−/−^ BM → WT), and (iv) WT mice reconstituted with WT BM (WT BM → WT). We confirmed lethal irradiation by measuring survival without BM reconstitution (fig. S1A), and BM reconstitution was confirmed by evaluating TNF-producing cells in circulation by flow cytometry [fluorescence-activated cell sorting (FACS)] analysis (fig. S1B). At 8 weeks after reconstitution, we intradermally inoculated the BM reconstituted mice with *S. aureus* and evaluated the skin lesion sizes and bacterial burdens. We found that mice that received BM cells from TNF^−/−^ mice (TNF^−/−^ BM → TNF^−/−^ and TNF^−/−^ BM → WT) had marked skin lesions and bacterial burdens, which were significantly reduced in mice that received BM from WT mice (WT BM → TNF^−/−^ and WT BM → WT) (Fig. 1, E and F). Moreover, there were no significant differences in lesion sizes or bacterial burden between WT or TNF^−/−^ mice that received BM cells from WT mice or between WT or TNF^−/−^ mice that received BM cells from TNF^−/−^ mice. These results indicated that TNF originating from BM-derived immune cells was important for immunity against *S. aureus* skin infections.

### Neutrophils are a predominant source of TNF during *S. aureus* skin infections

We next set out to determine the kinetics of TNF expression during the *S. aureus* skin infection to better understand which BM-derived immune cells contributed to TNF production. At baseline, WT mice had negligible TNF levels in the skin, which increased upon *S. aureus* infection on day 1 after infection, peaked on day 3 after infection, and subsided by day 7 after infection (Fig. 1G). Because we observed increased TNF production in the skin as early as day 1 after infection, we hypothesized that innate immune cells were a source of TNF during the *S. aureus* skin infection. We first quantified the myeloid cells (e.g., neutrophils, monocytes, and macrophages) in the skin during the *S. aureus* infection and found a notable peak in neutrophils on day 3 after infection, whereas monocytes and macrophages were lower in comparison (Fig. 1H). To identify whether myeloid cells were a source of TNF production, we performed flow cytometric analysis for intracellular TNF (see gating strategy in fig. S2) and found that neutrophils were a predominant source of TNF among BM-derived immune cells (CD45^+^) in the skin on day 3 after infection (Fig. 1, I and J). To a lesser extent, there were TNF-producing macrophages in the skin throughout the infection, whereas there were negligible numbers of TNF-producing monocytes.

### TNFR1 and TNFR2 signaling are both critical for host defense against *S. aureus* skin infections

**Fig. 2.**
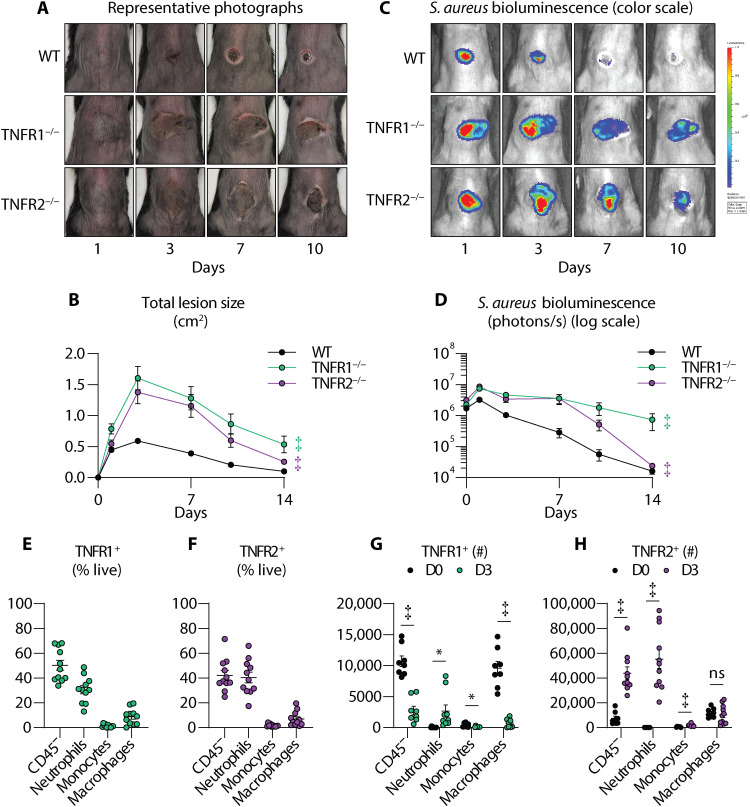
TNFR1 and TNFR2 signaling contribute to host defense against *S. aureus* skin infection. *S. aureus* (3 × 10^7^ CFU) skin infection was performed on WT and TNFR1-deficient (TNFR1^−/−^) and TNFR2-deficient (TNFR2^−/−^) mice (*n* ≥ 8 per group). (**A**) Representative photographs of skin lesions. (**B**) Mean total lesion size (cm^2^) ± SEM. (**C**) Representative in vivo BLI (photons/s) (log_10_ scale). (**D**) Mean total flux (photons/s) ± SEM. (**E** to **H**) WT skin tissue was harvested on day 0 and 3 after infection for flow cytometry analysis (*n* ≥ 9). (E) and (F) Mean TNFR1^+^ (E) or TNFR2^+^ (F) cells from live cells (%) ± SEM on day 3. (G) and (H) Total number of TNFR1^+^ (G) or TNFR2^+^ (H) neutrophils, monocytes, and macrophages ± SEM on day 3. Results are combined from at least two independent experiments. *P < 0.05, ‡*P* < 0.001, as calculated by a two-way ANOVA test (B) and (D) or a Student’s *t* test (two-tailed) (G) and (H).

TNF signals through TNFR1 and TNFR2, which have different downstream signaling pathways that dictate various biological outcomes, including inflammatory and host defense responses (*5*, *22*). To determine the relative contribution of TNFR1 and TNFR2 to host defense against *S. aureus* skin infections, we performed our intradermal *S. aureus* skin infection model in WT, TNFR1-deficient (TNFR1^−/−^), and TNFR2-deficient (TNFR2^−/−^) mice. We found that TNFR1^−/−^ and TNFR2^−/−^ mice developed significantly increased skin lesions and bacterial burdens compared to WT mice (Fig. 2, A to D). These data indicated that TNFR1 and TNFR2 were both important for TNF-mediated host defense against *S. aureus* skin infections.

### Neutrophils are a predominant TNFR1- and TNFR2-expressing cell type during *S. aureus* skin infection

Because both TNFR1 and TNFR2 were crucial for host defense, we next elucidated which cell types respond to TNF via TNFR1 and TNFR2 during the *S. aureus* skin infection. Therefore, we performed FACS analysis on single-cell suspensions from day 3 infected skin (see gating strategy in fig. S2) because this corresponded to peak TNF expression during the skin infection (Fig. 1G). By percentage of live cells, we found that skin-resident cells (CD45^−^) and neutrophils were the predominant TNFR1- and TNFR2-expressing cells in the day 3 infected skin, whereas macrophages and monocytes accounted for lesser amounts of the TNFR1- and TNFR2-expressing cells during the infection (Fig. 2, E and F). To determine whether TNFR expression was induced as a response to *S. aureus* infection, we compared TNFR1 and TNFR2 expression by FACS analysis from single-cell suspensions between uninfected skin (day 0) and day 3 infected skin. There was a marked reduction in TNFR1-expressing skin-resident cells, monocytes, and macrophages, whereas there was a significant increase in TNFR1-expressing neutrophils in the day 3 infected skin compared to uninfected skin (Fig. 2G). In contrast, TNFR2-expressing skin-resident cells, neutrophils, and monocytes were markedly increased, whereas macrophages had no change in the day 3 infected skin compared to uninfected skin (Fig. 2H). Collectively, populations of TNFR1- and TNFR2-expressing neutrophils were significantly increased in the *S. aureus*–infected skin.

### Neutrophil-intrinsic TNFR1 and TNFR2 signaling promotes protection against *S. aureus* skin infections

**Fig. 3.**
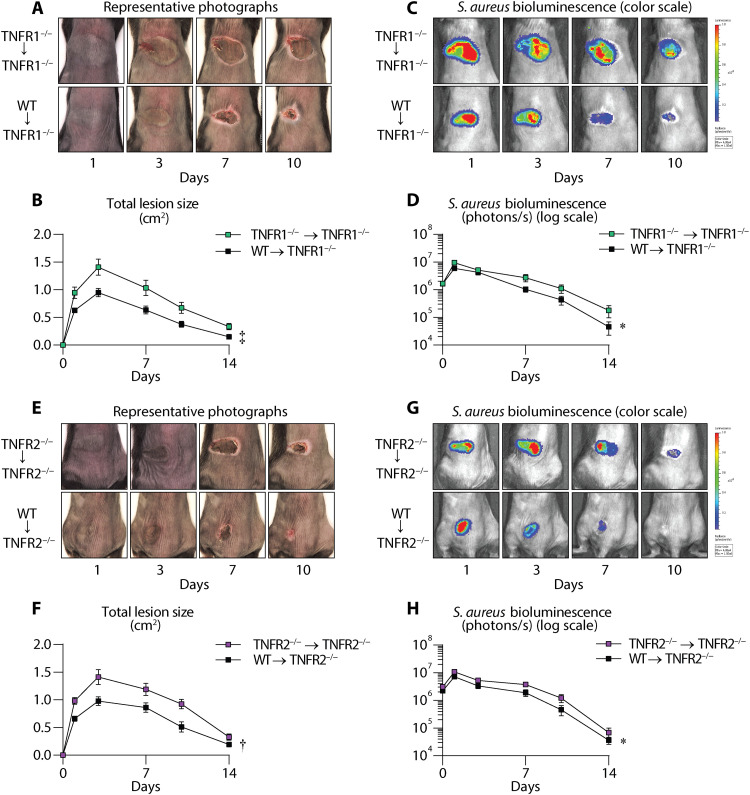
Neutrophil-intrinsic TNFR1 and TNFR2 signaling provide protection against *S. aureus* skin infections. Neutrophils from TNFR1^−/−^ or WT donor mice were adoptively transferred into (**A** to **D**) TNFR1^−/−^ mice or (**E** to **H**) neutrophils from TNFR2^−/−^ or WT donor mice into TNFR2^−/−^ mice (*n* ≥ 10 per group). After 2 hours, the recipient mice were infected intradermally with *S. aureus* (3 × 10^7^ CFU). (A) and (E) Representative photographs of skin lesions. (C) and (G) Representative in vivo BLI signals and total flux (photons/s) (log_10_ scale). (B) and (F) Mean total lesion size (cm^2^) ± SEM. (D) and (H) Mean total flux (photons/s) ± SEM. Results are combined from two independent experiments. *P < 0.05, †*P* < 0.01, ‡*P* < 0.001, as calculated by a two-way ANOVA test (B), (D), (F), and (H).

Neutrophil recruitment and abscess formation are critical for the resolution of *S. aureus* skin infections (*23*–*26*). Because there were markedly increased TNFR1- and TNFR2-expressing neutrophils in the infected skin, we hypothesized that neutrophil-intrinsic TNFR signaling was crucial for protection against *S. aureus* skin infections. Therefore, we performed adoptive transfer experiments whereby neutrophils purified from the BM of WT or TNFR1^−/−^ mice were adoptively transferred via retro-orbital intravenous injection into TNFR1^−/−^ mice 2 hours before *S. aureus* skin infection. In additional experiments, neutrophils from WT or TNFR2^−/−^ mice were adoptively transferred into TNFR2^−/−^ mice. The viability (>94%) and purity (>92%) of the neutrophils were confirmed before proceeding with the adoptive transfer experiments (fig. S3, A to C). Adoptive transfer of WT neutrophils into TNFR1^−/−^ mice (WT → TNFR1^−/−^) led to significantly reduced lesion sizes and bacterial burden compared to adoptive transfer of TNFR1^−/−^ neutrophils into TNFR1^−/−^ mice (TNFR1^−/−^ → TNFR1^−/−^) (Fig. 3, A to D). Similarly, adoptive transfer of WT neutrophils into TNFR2^−/−^ mice (WT → TNFR2^−/−^) caused markedly decreased skin lesion sizes and bacterial burden compared to adoptive transfer of TNFR2^−/−^ neutrophils into TNFR2^−/−^ mice (TNFR2^−/−^ → TNFR2^−/−^) (Fig. 3, E and F). Together, neutrophil-intrinsic TNFR1 and TNFR2 signaling contributed to immunity against *S. aureus* skin infections.

### TNFR1 signaling promotes neutrophilic abscess formation in the skin

**Fig. 4.**
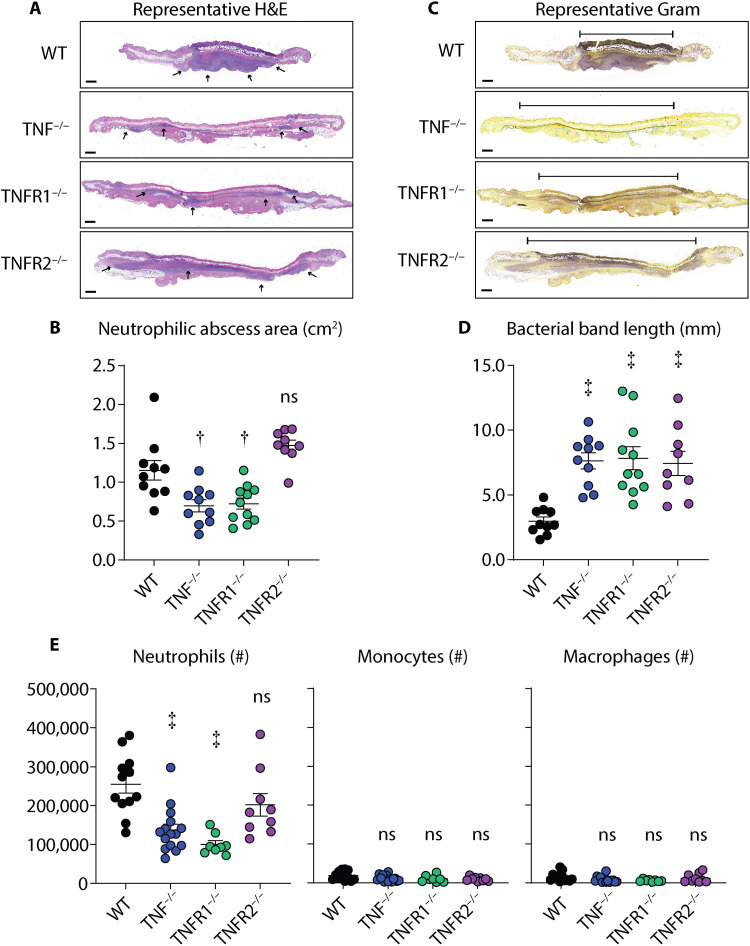
TNFR1 promotes neutrophilic abscess formation and recruitment during *S. aureus* skin infections. *S. aureus* (3 × 10^7^ CFU) skin infections were performed on WT, TNF^−/−^, TNFR1^−/−^, and TNFR2^−/−^ mice and the infected skin tissue was harvested on day 3 after infection for histology (*n* ≥ 8 per group) and for flow cytometry analysis (*n* ≥ 8 per group). (**A**) Representative H&E skin sections (scale bars, 500 μm). Black arrows indicate the region of the horizontal area of neutrophilic abscess. (**B**) Mean neutrophilic abscess area (cm^2^) ± SEM. (**C**) Representative Gram stain skin sections (scale bars, 500 μm). (**D**) Mean bacterial band width (mm) ± SEM. (**E**) Total number of neutrophils, monocytes, and macrophages ± SEM from *S. aureus*–infected skin on day 3 after infection. Results are combined from at least two independent experiments. †*P* < 0.01, ‡*P* < 0.001, as calculated by a one-way ANOVA multiple comparisons test.

TNF has previously been reported to promote neutrophil recruitment to sites of contact hypersensitivity–induced skin inflammation (*19*). Because neutrophilic abscess formation is a hallmark of immunity against *S. aureus* skin infections (*24*–*26*), we next asked whether TNF and TNFR signaling affected neutrophil recruitment during the *S. aureus* skin infection. To this end, we compared neutrophilic abscess formation on day 3 infected skin from hematoxylin and eosin (H&E)–stained skin sections of WT, TNF^−/−^, TNFR1^−/−^, and TNFR2^−/−^ mice. Unexpectedly, we found that the neutrophilic abscess area (as denoted by the black arrows, Fig. 4A) was abrogated in TNF^−/−^ and TNFR1^−/−^ but not TNFR2^−/−^ mice compared to WT mice (Fig. 4, A and B). In contrast, analysis of Gram-stained skin sections showed that TNF^−/−^, TNFR1^−/−^, and TNFR2^−/−^ mice all had significantly increased bacterial band lengths compared to WT mice (Fig. 4, C and D). To confirm our results, we performed FACS analysis from day 3 infected skin (see gating strategy in fig. S4) and found a similar pattern as our neutrophilic abscess area results whereby neutrophil numbers were markedly decreased in the skin of TNF^−/−^ and TNFR1^−/−^ but not TNFR2^−/−^ mice compared to WT mice (Fig. 4E). These findings were specific to neutrophils, as monocytes and macrophages had comparable levels between WT mice and TNF^−/−^, TNFR1^−/−^, and TNFR2^−/−^ mice (Fig. 4E). Collectively, our data suggest that TNFR1 signaling promotes neutrophil trafficking and ensuing neutrophilic abscess formation for optimal host defense against *S. aureus* skin infections.

### Neutrophil extracellular traps foster protection against *S. aureus* skin infections

**Fig. 5.**
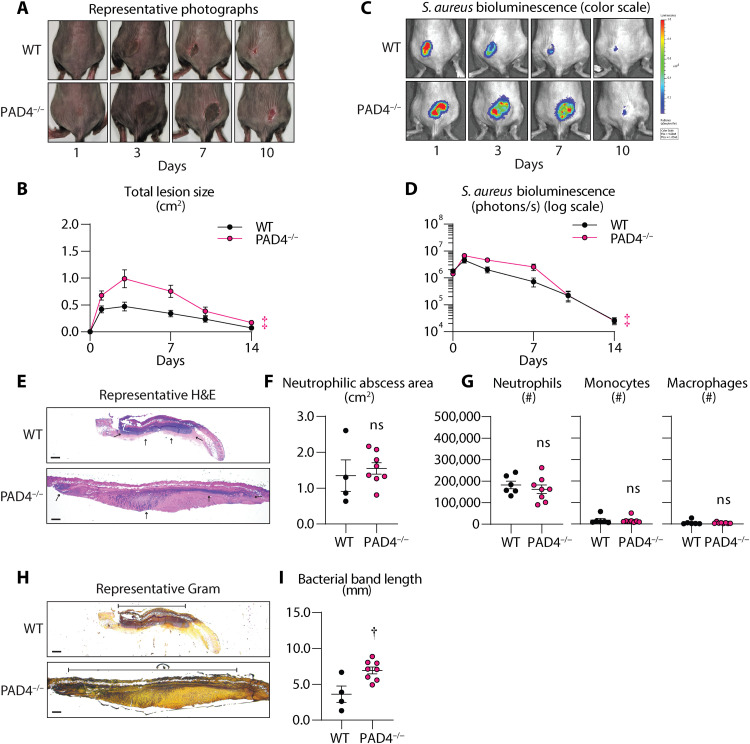
NETs contribute to host defense against *S. aureus* skin infection. *S. aureus* (3 × 10^7^ CFU) skin infection was performed on WT and PAD4-deficient (PAD4^−/−^) (*n* ≥ 8 per group). (**A**) Representative photographs of skin lesions. (**B**) Mean total lesion size (cm^2^) ± SEM. (**C**) Representative in vivo BLI signals and total flux (photons/s) (log_10_ scale). (**D**) Mean total flux (photons/s) ± SEM. (**E** to **I**) Skin tissue was harvested on day 3 after infection from WT and PAD4^−/−^ mice for histology (*n* ≥ 4 per group) and for flow cytometry analysis (*n* ≥ 6 per group). (E) Representative H&E skin sections (scale bars, 500 μm). Black arrows indicate the region of the horizontal area of neutrophilic abscess. (F) Mean neutrophilic abscess area (cm^2^) ± SEM. (G) Total number of neutrophils, monocytes, and macrophages ± SEM. (H) Representative Gram stain skin sections (scale bars, 500 μm). (I) Mean bacterial band width (mm) ± SEM. Results are representative of at least two independent experiments. †*P* < 0.01, ‡*P* < 0.001, as calculated by a two-way ANOVA test (B) and (D) or a Student’s *t* test (two-tailed) (F), (G), and (I).

Neutrophil extracellular traps (NETs) form upon neutrophil exposure to an external stimulus, including TNF and *S. aureus* (*27*–*29*), which induces the release of decondensed chromatin decorated with antimicrobial proteins to trap and kill microbes (*30*). However, the role of NETs in host defense against *S. aureus* skin infections is not entirely clear. To determine whether NET formation is protective during *S. aureus* skin infections, mice deficient in peptidylarginine deiminase 4 (PAD4^−/−^), which facilitates chromatin decondensation and subsequent NET formation (*31*), were intradermally inoculated with *S. aureus*. We found that PAD4^−/−^ mice developed significantly increased skin lesion sizes and bacterial burden relative to WT mice (Fig. 5, A to D), which was most pronounced at time points when neutrophils migrated into the skin (Fig. 1H). PAD4^−/−^ mice had comparable neutrophilic abscess areas and neutrophil recruitment in the skin compared to WT mice (Fig. 5, E to G) despite a larger bacterial band length (Fig. 5, H and I). Together, these data suggested that PAD4-dependent NET formation is an important host defense mechanism against *S. aureus* skin infection.

### TNFR2 signaling promotes NET formation in the skin

**Fig. 6.**
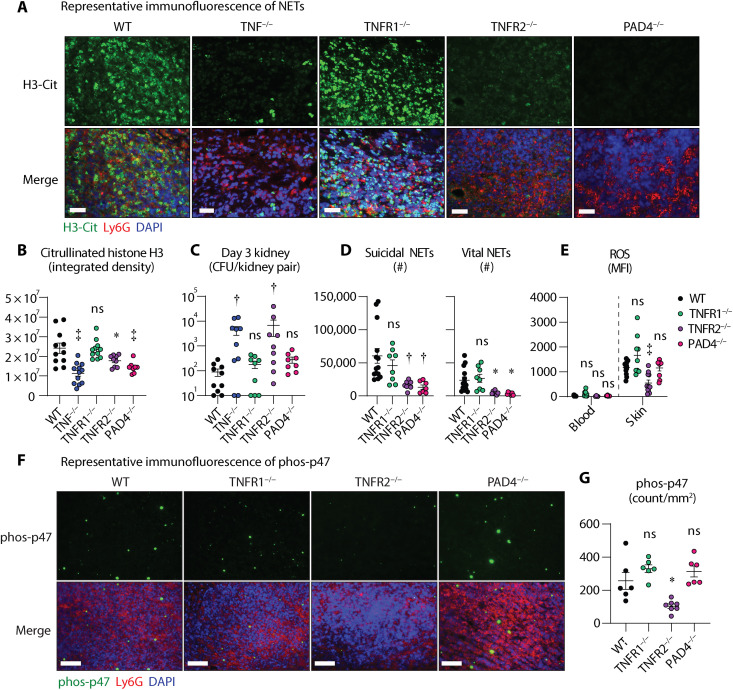
TNFR2 is crucial for NET formation, NOX2 activation, and ROS production during *S. aureus* skin infections. *S. aureus* (3 × 10^7^ CFU) skin infections were performed on WT, TNF^−/−^, TNFR1^−/−^, TNFR2^−/−^, and PAD4^−/−^ mice and the infected skin and kidneys were harvested on day 3 after infection for histology (*n* ≥ 6 per group), flow cytometry analysis (*n* ≥ 8 per group), and CFU counts (*n* ≥ 8 per group). (**A**) Representative immunofluorescence microscopy sections labeled with anti–H3-Cit (green), anti-Ly6G (red), and 4′,6-diamidino-2-phenylindole (DAPI) (blue) (scale bars, 100 μm). (**B**) Integrated density of H3-Cit ± SEM. (**C**) Mean colony forming units (CFU) per kidney pair (log scale) ± SEM. (**D**) Total number of suicidal and vital NETs ± SEM. (**E**) Mean of ROS mean fluorescence intensity (MFI) ± SEM in neutrophils. (**F**) Representative immunofluorescence microscopy sections labeled with anti–phosphorylated p47 (phos-p47) (green), anti-Ly6G (red), and DAPI (blue) (scale bars, 100 μm). (**G**) Mean count of phos-p47 puncta (count/mm^2^) ± SEM. Results are combined from at least two independent experiments. *P < 0.05, †*P* < 0.01, ‡*P* < 0.001, as calculated by one-way ANOVA multiple comparisons test (B) to (E) and (G).

Because TNF facilitates NET formation in vitro (*27*, *32*), we hypothesized that TNF signaling was important for NET formation during *S. aureus* skin infection. To determine the role of TNF and the differential contributions of TNFR1 and TNFR2 in NET formation during the *S. aureus* skin infection, we examined day 3 infected skin sections from WT, TNF^−/−^, TNFR1^−/−^, and TNFR2^−/−^ mice for citrullinated histone 3 (H3-Cit) and Ly6G, which are established markers for NET formation (*32*–*34*). In addition, we included PAD4^−/−^ mice as a negative control for NET formation. As expected, infected skin from WT mice exhibited robust NET formation as indicated by intense H3-Cit stain in proximity to Ly6G, which was markedly diminished in PAD4^−/−^ mice (Fig. 6, A and B). Notably, infected skin from TNF^−/−^ and TNFR2^−/−^, but not TNFR1^−/−^, mice showed markedly abrogated H3-Cit stain compared to WT mice (Fig. 6A). H3-Cit intensity was quantified by integrated fluorescence intensity, which showed a significant reduction in TNF^−/−^, TNFR2^−/−^, and PAD4^−/−^ mice but not in TNFR1^−/−^ mice compared to WT mice (Fig. 6B). Because NETs immobilize and control spreading of pathogens (*35*, *36*), we next asked whether TNF-mediated NET formation prevented bacterial dissemination from the skin to kidneys on day 3 of the infection. Unexpectedly, while we saw significantly higher bacterial burdens in the kidneys of TNF^−/−^ and TNFR2^−/−^ mice, TNFR1^−/−^ and PAD4^−/−^ had comparable CFU counts in the kidney compared to WT mice (Fig. 6C). Collectively, these results indicated that TNFR2 signaling promotes NET formation and prevents bacterial dissemination during *S. aureus* skin infections.

Depending on the type and duration of the external stimuli, neutrophils form either suicidal or vital NETs (*37*). Suicidal NET formation involves cell membrane lysis that leads to cell death, whereas vital NET formation relies on the release of decondensed chromatin from vesicles, leaving the cell membrane intact for cell survival (*37*, *38*). However, it is unknown which type of NETs form during *S. aureus* infections in the skin or how TNF signaling influences suicidal or vital NET formation. To evaluate the levels of suicidal and vital NET formation in the skin, neutrophils from the skin of WT, TNFR1^−/−^, TNFR2^−/−^, and PAD4^−/−^ mice were assessed by FACS analysis as previously described (see gating strategy in fig. S5A) (*39*). While suicidal and vital NET formation was comparable between WT and TNFR1^−/−^ mice, both suicidal and vital NET formation were significantly diminished in TNFR2^−/−^ and PAD4^−/−^ mice compared to WT mice (Fig. 6D and fig. S5B). In vitro stimulation of WT, TNFR1^−/−^, and TNFR2^−/−^ neutrophils with phorbol myristate acetate (PMA) or live *S. aureus* induced vital but not suicidal NET formation (fig. S6, A to C). Consistent with our in vivo findings, TNFR2^−/−^ neutrophils cocultured with *S. aureus* had significantly reduced vital NET formation compared to WT neutrophils (fig. S6C). These findings indicated that TNFR2 signaling was crucial for both suicidal and vital NET formation in response to the *S. aureus* skin infection.

### TNFR2 signaling initiates reactive oxygen species generation via NADPH oxidase 2 activation in neutrophils

Suicidal NET formation is dependent on the generation of reactive oxygen species (ROS) in neutrophils (*37*, *40*). Given our findings that TNFR2 is important for suicidal NET formation, we hypothesized that TNFR2 signaling induced ROS production in neutrophils during *S. aureus* skin infections. Thus, we used FACS analysis to examine the ROS levels in neutrophils from day 3 infected skin and blood of WT, TNFR1^−/−^, TNFR2^−/−^, and PAD4^−/−^ mice. ROS production was negligible in circulating neutrophils in all the tested groups, whereas upon infiltration into the infected skin, WT neutrophils had robust ROS production that was markedly decreased in TNFR2^−/−^ neutrophils but not in TNFR1^−/−^ andPAD4^−/−^ neutrophils (Fig. 6E). In vitro stimulation of neutrophils with live *S. aureus* induced ROS production (fig. S7), which was dependent on both TNFR1 and TNFR2 signaling (fig. S7B).

Neutrophils express NADPH oxidase 2 (NOX2) that enables robust production of ROS for antimicrobial host defense (*41*, *42*). NOX2 is a multicomponent enzymatic complex that becomes activated upon assembly of cytosolic components (p47_phox_, p67_phox_, p40_phox_, and Rac2) with its transmembrane proteins (p22_phox_ and gp91_phox_) (*43*, *44*). Phosphorylation of p47_phox_ is critical for the assembly of the cytosolic subunits and mobilization of NOX2 to initiate ROS production (*44*). To determine whether ROS production in neutrophils involved NOX2 activation downstream of TNFR2 signaling, we performed immunofluorescent microscopy for phosphorylated p47_phox_ (phos-p47) on day 3 infected skin sections from WT, TNFR1^−/−^, TNFR2^−/−^, and PAD4^−/−^ mice. Consistent with our ROS findings, WT infected skin had abundant phos-p47 puncta in the neutrophilic abscess that was significantly reduced in TNFR2^−/−^ skin but not in TNFR1^−/−^ and PAD4^−/−^ skin (Fig. 6, F and G). These data indicated that TNFR2 signaling induced ROS production via NOX2 activation in neutrophils during *S. aureus* skin infections.

### A TNFR2 agonist has efficacy against *S. aureus* skin infections in mice

**Fig. 7.**
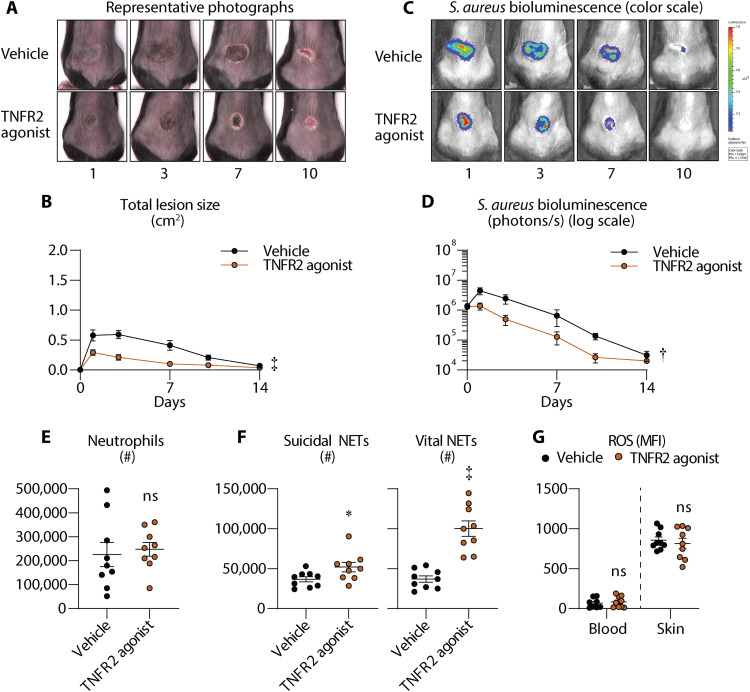
TNFR2 agonist has efficacy against *S. aureus* skin infections in mice. Mice were treated with a single dose of vehicle (sterile PBS) or TNFR2 agonist [8 mg/kg, intraperitoneally (i.p.)] (*n* ≥ 7 mice per group) at 4 hours after *S. aureus* (3 × 10^7^ CFU) intradermal skin inoculation. (**A**) Representative photographs of skin lesions. (**B**) Mean total lesion size (cm^2^) ± SEM. (**C**) Representative in vivo BLI. (**D**) Mean total flux (photons/s) ± SEM. (**E** to **G**) Infected skin was harvested on day 3 after infection from vehicle-treated or TNFR2 agonist–treated mice for flow cytometry analysis (*n* ≥ 9 per group). (E) Total number of neutrophils ± SEM. (F) Total number of suicidal and vital NETs ± SEM. (G) Mean ROS mean fluorescence intensity (MFI) ± SEM in neutrophils. *P < 0.05, †*P* < 0.01, ‡*P* < 0.001, as calculated by a two-way ANOVA test (B) and (D) or a Student’s *t* test (two-tailed) (E) to (G). Data are compilations of at least two independent experiments.

Because TNF inhibitors promote *S. aureus* skin colonization and infection in patients (*13*, *14*), and TNFR2 signaling drives neutrophil NET formation and NOX2-mediated ROS production, we postulated that TNFR2 agonism would enhance host defense against *S. aureus* skin infections via increased neutrophil activation. To this end, we therapeutically treated WT mice with a single intraperitoneal dose of a TNFR2 agonist (*45*) or vehicle 4 hours following the *S. aureus* intradermal inoculation. The TNFR2 agonist treatment resulted in markedly reduced skin lesion sizes (Fig. 7, A and B) and bacterial burdens (Fig. 7, C and D) as compared to vehicle-treated mice. The efficacy of the TNFR2 agonist against the *S. aureus* skin infection was not due to direct antistaphylococcal activity, as there were no differences in the growth curves of *S. aureus* incubated with varying concentrations of the TNFR2 agonist (80, 160, and 320 μg/ml) and vehicle control (fig. S6A). To note, the 160 μg/ml TNFR2 agonist concentration corresponded to the equivalent dose administered to the mice.

To evaluate whether the TNFR2 agonist altered neutrophil recruitment and/or function, we measured neutrophil levels, suicidal and vital NET formation, and neutrophil ROS production following TNFR2 agonist or vehicle treatment in day 3 infected skin. While there were no significant differences in the number of neutrophils in the skin (Fig. 7E), we observed a marked increase in suicidal and vital NET formation in TNFR2 agonist–treated mice compared to vehicle-treated mice (Fig. 7F). Despite an increase in suicidal NET formation, we found no differences in the ROS production between TNFR2 agonist– and vehicle-treated neutrophils in circulation and skin (Fig. 7G). Together, TNFR2 agonist treatment promoted host defense against *S. aureus* skin infections by a mechanism that involved increased suicidal and vital NET formation.

### TNFR2 agonist treatment has efficacy against *Pseudomonas aeruginosa* skin infections in mice

**Fig. 8.**
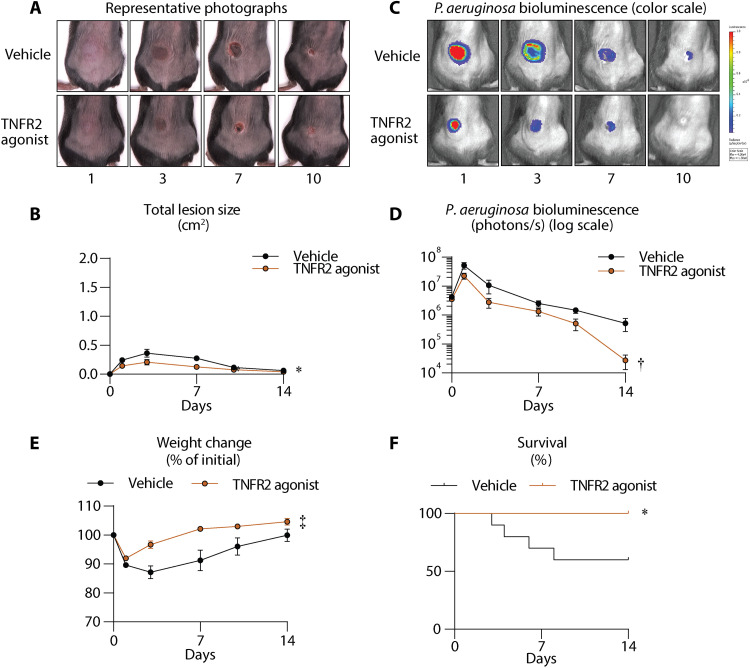
TNFR2 agonist has efficacy against *P. aeruginosa* skin infections in mice. Mice were treated with a single dose of vehicle (sterile PBS) or TNFR2 agonist (8 mg/kg, i.p.) (*n* ≥ 10 mice per group) at 4 hours after *P. aeruginosa* (1 × 10^6^ CFU) intradermal skin inoculation. (**A**) Representative photographs of skin lesions of *P. aeruginosa* infection. (**B**) Mean total lesion size (cm^2^) ± SEM of *P. aeruginosa* infection. (**C**) Representative in vivo bioluminescence imaging (BLI) of *P. aeruginosa* infection. (**D**) Mean total flux (photons/s) ± SEM of *P. aeruginosa* infection. (**E**) Mean weight change (% of initial) ± SEM. (**F**) Kaplan-Meier survival curves of vehicle- and TNFR2 agonist–treated mice after *P. aeruginosa* intradermal infection. *P < 0.05, †*P* < 0.01, ‡*P* < 0.001, as calculated by a two-way ANOVA test (B), (D), and (F). Data are a compilation of at least two independent experiments.

Given the efficacy of the TNFR2 agonist against *S. aureus*, a Gram-positive skin pathogen, we next examined whether the antimicrobial efficacy extended to the Gram-negative skin pathogen, *Pseudomonas aeruginosa* (*4*, *46*). Therefore, we performed our intradermal infection model with a bioluminescent *P. aeruginosa* strain [Xen41 (*47*)] in WT mice. A single dose of the TNFR2 agonist or vehicle was administered intraperitoneally 4 hours after infection, and lesion sizes and in vivo BLI were longitudinally measured over 14 days. Similar to the *S. aureus* skin infection, TNFR2 agonist–treated mice had substantially decreased skin lesion sizes (Fig. 8, A and B) and reduced bacterial burden compared to vehicle-treated mice (Fig. 8, C and D). Moreover, the *P. aeruginosa* skin infection caused marked reductions in weight and survival in the vehicle-treated mice, which were significantly improved in the TNFR2 agonist–treated mice (Fig. 8, E and F). The efficacy of the TNFR2 agonist against the *P. aeruginosa* skin infection was not due to direct antibacterial activity, as there were no differences in the growth curves of *P. aeruginosa* incubated with varying concentrations of the TNFR2 agonist (80, 160, and 320 μg/ml) and vehicle control (fig. S6B). These results indicated that the TNFR2 agonist had therapeutic efficacy against *P. aeruginosa* skin infections.

## DISCUSSION

**Fig. 9.**
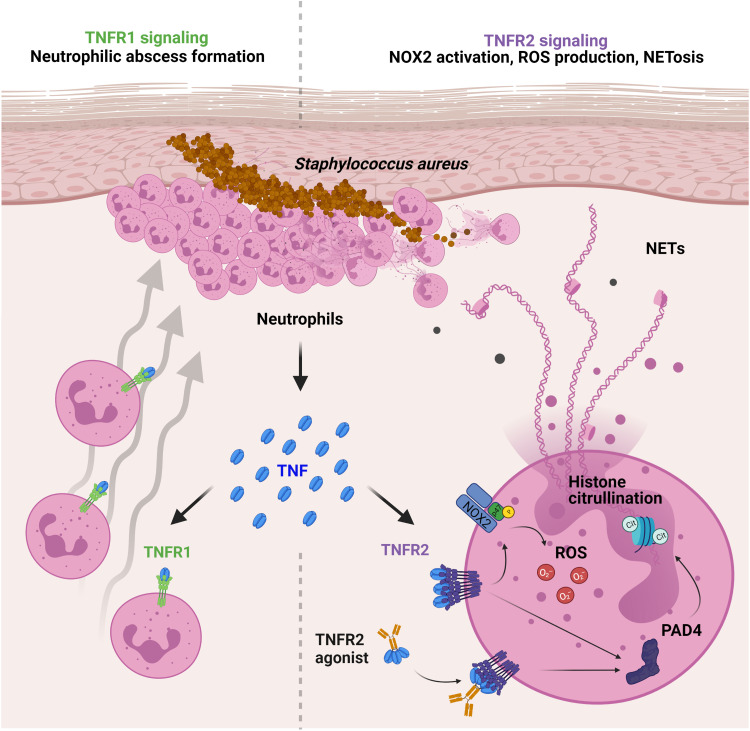
Model of TNFR-mediated neutrophil responses against *S. aureus* skin infection. *S. aureus* intradermal infections result in TNF expression by recruited neutrophils in the skin. TNF acts directly on neutrophils by TNFR1 to increase their recruitment to the skin, whereas TNFR2 promoted NET formation, ROS production, and NOX2 activation. TNFR2 agonist treatment improved the immune response against bacterial skin infections by enhancing NET formation in the infected skin.

TNF is a pleiotropic cytokine involved in inflammatory conditions and host defense against infection (*48*). However, the role of TNF in the protection against *S. aureus* skin infections is not well defined. Herein, we found that TNF had a crucial host defense role against *S. aureus* in the skin, with neutrophils as both a major TNF-producing cell and a predominant TNFR1- and TNFR2-expressing cell during the *S. aureus* skin infection (Fig. 9). Furthermore, TNFR1 and TNFR2 had critical and nonredundant roles in host defense that included neutrophil recruitment and abscess formation, and neutrophil activation (e.g., ROS production and NET formation), respectively. Targeting TNFR2 with a TNFR2 agonist enhanced NET formation and protected against clinically relevant Gram-positive and Gram-negative skin pathogens (e.g., *S. aureus* and *P. aeruginosa*). Collectively, these findings provide new and important insights into TNF biology and function in skin immunity.

First, we found that TNF had an important host defense role against *S. aureus* in the skin. This finding provides a potential explanation for the increased susceptibility of patients on TNF inhibitors to develop *S. aureus* skin infections (*13*, *49*) and aligns with reports by our group and others that TNF responses are protective against *S. aureus* at other infection sites, including orthopedic implant–associated infections (*50*), bacteremia (*51*), and brain abscess infections (*52*, *53*). In addition, we found that BM cell–derived TNF promoted bacterial clearance, with neutrophils as a major producer of TNF during the *S. aureus* infection in the skin. Similarly, neutrophils produced TNF in response to *Mycobacterium tuberculosis* infection (*54*), and macrophage/neutrophil-derived TNF was critical for protection and survival against a systemic *Listeria monocytogenes* infection (*55*), indicating that myeloid cell–derived TNF is a relevant immune response against bacterial infections. However, the differential contributions of TNF produced by myeloid cells and other immune cells for host defense against *S. aureus* will be the focus of future work.

We found that TNFR1 and TNFR2 had nonredundant contributions to immunity against *S. aureus* skin infections, which involved neutrophil-intrinsic signaling. For instance, we observed that TNFR1 signaling affected neutrophil recruitment and neutrophilic abscess formation at the site of the *S. aureus* skin infection. A potential explanation for this finding is that TNFR1 engagement directly primed neutrophils to extravasate into the skin, as was previously reported in a mouse model of contact hypersensitivity (*19*). In contrast, Miller *et al.* (*56*) showed no immune impairment in TNFR1-deficient mice during a *S. aureus* skin infection. This discrepancy may be due to differences in the *S. aureus* strains [e.g., MRSA versus methicillin-susceptible *S. aureus* (MSSA)], which include a greater ability of MRSA to intracellularly survive and replicate in macrophages (*57*), increased hemolytic activity (*58*), and elevated expression of extracellular enzymes (e.g., aureolysin, serine proteases, thermonuclease, and lipase) and toxins (e.g., hemolysins) that manipulate host innate immunity compared to MSSA (*59*). This may be particularly relevant as *S. aureus* protein A (SpA) binds and activates TNFR1 (*60*), including on human neutrophils to induce interleukin-8 (IL-8), TNF, and macrophage inflammatory protein–1α expression (*61*). Furthermore, SpA promotes TNFR1 shedding to dampen inflammatory outcomes (*62*, *63*), which may explain the reduced TNFR1 expression on nonimmune cells and macrophages during the *S. aureus* skin infection compared to naïve skin. However, whether SpA influences neutrophilic abscess formation via TNFR1 during *S. aureus* skin infections warrants further investigation.

We found that TNFR2 promoted *S. aureus* clearance in the skin, in a mechanism that involved neutrophil activation. This was unexpected because TNFR2 is associated with immunosuppressive functions in other immune cells (*64*, *65*). For example, TNFR2 drives T_reg_ differentiation, expansion, and anti-inflammatory responses in models of autoimmunity (*8*, *18*, *66*) and activates myeloid-derived suppressor cell (MDSC) (*67*) and natural killer (NK) cell (*68*) suppressor functions in the tumor microenvironment, suggesting that TNFR2 signaling has cell-specific and contextual-dependent immunological functions. Whether TNFR2 signaling in T_regs_, MDSC, or NK cells influences host defense during *S. aureus* skin infections will be examined in future studies. Another finding was that TNFR2 but not TNFR1 mediated NOX2 activation and ROS production in the in vivo neutrophils. However, both TNFR1 and TNFR2 contributed to ROS production in the in vitro neutrophils when cocultured with *S. aureus*. This was unanticipated because TNFR1 harbors a cytosolic domain, which is not shared with TNFR2, that allows direct interactions with RFK to activate the NOX2 complex (*69*). Furthermore, in a preclinical model of colorectal tumors, TNFR1 signaling enhanced oxidative stress and tumor cell death by a mechanism that involved NOX-mediated ROS production (*70*). A possible explanation for our findings is that the cellular microenvironment of the neutrophil-rich abscess favors transmembrane TNF (mTNF) signaling rather than soluble TNF on neutrophils, as TNFR2 activation is dependent on receptor aggregation and has preferential sensitivity to mTNF present on cell surfaces (*71*, *72*). Elucidating the mechanism by which TNFR2 engages NOX2 in a TNFR1-independent manner will be investigated in our future work.

We found that TNFR2, but not TNFR1, was responsible for in vivo and in vitro NET formation in response to *S. aureus* exposure. Our observations align with previous in vitro studies that showed TNFR2-mediated pro-NETotic activity in human neutrophils (*73*) and mTNF-induced caspase- and death domain–independent cell death via mitochondrial ROS production (*74*). Another interesting finding was that TNFR2 controlled PAD4-dependent suicidal and vital NET formation during the *S. aureus* skin infection. Because suicidal NETs are ROS dependent (*75*, *76*), our observation that TNFR2 regulates NOX2 activation and ROS production provides an explanation for the dependence of suicidal NET formation on TNFR2 signaling. In contrast, vital NETosis is independent of ROS production by NOX (*29*, *36*). Our finding that TNFR2 regulates vital NETosis aligns with a previous study that showed NOX activity is dispensable in TNF-stimulated NET formation in vitro (*77*). Understanding the in vivo mechanisms of TNFR2-mediated suicidal and vital NET formation during *S. aureus* skin infections will be the focus of future work.

We found that TNFR2 but not PAD4 was critical for preventing systemic dissemination of the *S. aureus* skin infection, suggesting that NETs were dispensable for the containment of *S. aureus* in the skin. These results were unexpected because Yipp. *et al.* (*36*) found that NETs were essential for limiting *S. aureus* dissemination from the skin. This discrepancy may be explained by the differences in the skin infection models used (e.g., intradermal versus subcutaneous) and/or the methods to inhibit in vivo NET formation [e.g., PAD4-deficient mice versus deoxyribonuclease (DNase) treatment]. Regardless, our findings indicated that TNFR2 is crucial in controlling *S. aureus* spread from the skin in a mechanism independent of NET formation.

Last, we found that TNFR2 agonism acted as a novel immunotherapeutic strategy against bacterial skin infections, providing an important translational component to our findings. This antimicrobial efficacy was unanticipated because previous studies have used TNFR2 agonists to dampen pathogenic inflammatory responses in the context of neurodegeneration (*78*, *79*), cancer (*80*), and acute graft-versus-host disease (*45*, *81*), including the TNFR2 agonist used in this study (*45*). We found that the TNFR2 agonist enhanced NET formation, in particular vital NETosis, which was associated with improved *S. aureus* clearance. Whether efficacy of the TNFR2 agonist efficacy is dependent on either suicidal or vital NETosis or another mechanism will be interrogated in our future work. Because TNFR2 agonist–treated mice did not have increased ROS in neutrophils, it is possible that the TNFR2 agonist acts by priming NOX in neutrophils before *S. aureus* exposure in the skin. Thus, primed neutrophils can produce ROS more quickly in response to infection compared to vehicle-treated neutrophils. Furthermore, TNFR2 agonism showed activity against Gram-positive and Gram-negative skin pathogens, indicating that this strategy has potential as a broad-spectrum antibacterial therapy. We also predict that TNFR1 agonism may act as a therapeutic target alone or additively in combination with the TNFR2 agonist, because our data suggest that TNFR1 and TNFR2 have nonredundant roles in inducing neutrophil recruitment and function, respectively. In addition, our findings have important clinical implications beyond bacterial infections, specifically in the pathogenesis of inflammatory diseases exacerbated by NET formation (*82*, *83*) and provide a potential mechanistic explanation for the efficacy of TNF inhibitors to dampen inflammation in these conditions. However, additional studies are warranted to determine whether TNFR2 agonism promotes protection against additional pathogens and infections at sites other than the skin.

There are several limitations. First, we did not interrogate the role of TNFR signaling on T cells, especially because TNF promotes activation and proliferation of effector and memory T cells (*84*, *85*). In addition to TNF, lymphotoxin α (LTα) binds to both TNFR1 and TNFR2 (*86*). Therefore, we cannot rule out a contribution of LTα to host defense against *S. aureus* skin infections. Furthermore, we focused our attention on NOX2-mediated ROS production in neutrophils and did not examine the contribution of mitochondrial-derived ROS in neutrophils, as was previously reported to promote suicidal NET formation during a systemic *S. aureus* infection (*39*). Last, we only tested the TNFR2 agonist against a single strain of *S. aureus* and *P. aeruginosa*, limiting the conclusions of these results.

Together, neutrophil-intrinsic TNFR1 and TNFR2 signaling orchestrated host defense against *S. aureus* skin infections in a mechanism that included neutrophil recruitment, NOX2-mediated ROS production, and NET formation. Furthermore, TNFR2 agonist treatment improved clearance of *S. aureus* and *P. aeruginosa* skin infections, which involved increased NET formation at the infection site. These findings provide important mechanistic insights and feasibility for agonistic targeting of TNFR2 as a novel immunotherapeutic strategy to treat antibiotic-resistant bacterial infections.

## MATERIALS AND METHODS

### Experimental design

The study’s objective was to determine the role of TNF and the TNFRs in host defense against *S. aureus* skin infections and whether this pathway could be therapeutically targeted as a novel host-directed strategy against bacterial infections. We used an intradermal *S. aureus* skin infection mouse model whereby bacterial burden and skin lesions were longitudinally measured in vivo for 14 days in WT mice or TNF-, TNFR1-, TNFR2-, and PAD4-deficient mice. In additional experiments, WT mice were infected intradermally with *S. aureus* or *P. aeruginosa* and therapeutically treated with vehicle or TNFR2 agonist. The maximum neutrophil influx and TNF expression in WT mice was observed 3 days after infection. Therefore, 3 days post-infection skin was used to compare outcomes between comparison groups. Cells isolated from infected mouse skin were profiled by multiparametric flow cytometry to identify changes in cell populations, cytokine and receptor expression, ROS production, and NET formation. Furthermore, skin biopsies from infected mice were analyzed by histology for neutrophilic abscess formation and bacterial band length, and immunohistochemistry to quantify NET formation and NOX2 activation. The group sizes for each mouse strain included at least four mice per group with at least two iterations to confirm results, which was sufficient to ensure statistical significance, as previously described (*15*, *84*). Each experimental group was paired with a respective control group to control for variation in bacterial inoculum between independent experiments. Outliers were identified and excluded by Grubb’s test with Prism software (GraphPad 9 Software, La Jolla, CA). Animal caretakers and researchers were not blinded to the study groups. All animal studies were approved by the Johns Hopkins University Animal Care and Use Committee.

### Mouse model of intradermal bacterial skin infection

A mouse model of *S. aureus* intradermal infection was performed as previously described (*15*, *16*, *84*). Briefly, the dorsal skin of anesthetized mice (2% isoflurane) was shaved and a 100-μl volume of phosphate-buffered saline (PBS) containing 3 × 10^7^ CFU of USA300 LAC::lux or 1 × 10^6^ CFU of Xen41 was intradermally injected. Total lesion size (cm^2^) was measured from digital photographs of the infected skin of anesthetized mice (2% isoflurane) by using the image analysis software program ImageJ (https://imagej.nih.gov/ij/) with a millimeter ruler as a reference.

### Bacterial strains

The bioluminescent *S. aureus* USA300 LAC::lux was derived from the parent *S. aureus* strain USA300 LAC, a well-described CA-MRSA clinical isolate that was obtained from an *S. aureus* SSTI outbreak in the Los Angeles County (LAC) Jail (*15*, *84*, *87*). The bioluminescent *P. aeruginosa* strain Xen41 (PerkinElmer) was obtained from the parental strain PAO1 (*88*). USA300 LAC::lux and Xen41 have a modified luxABCDE operon from the bacterial insect pathogen, *Photorhabdus luminescens*, that the emission of blue-green light from live metabolically active bacteria is maintained in all progeny.

### Bacterial preparation

Bacterial strains were prepared as previously described (*16*, *84*). *S. aureus* USA300 LAC::lux bacteria were streaked onto a tryptic soy agar (TSA) plate [tryptic soy broth (TSB) plus 1.5% bacto agar] and grown overnight at 37°C in a bacterial incubator. Single colonies were picked and cultured in TSB at 37°C in a shaking incubator (240 rpm) overnight (18 hours), followed by a 1:50 subculture at 37°C for 2 hours to obtain mid-logarithmic phase bacteria. *P. aeruginosa* Xen41 bacteria were streaked onto a Luria-Bertani (LB) plate (LB broth and 1.5% bacto agar) and grown overnight at 37°C in a bacterial incubator. Single colonies of *P. aeruginosa* strain were grown overnight in LB broth at 37°C shaking at 240 rpm, then diluted 1:50, and grown for 2.5 hours to obtain midlogarithmic growth phase bacteria. The bacteria were pelleted, washed three times, and resuspended in sterile PBS at the indicated concentrations for each bacterium below. The absorbance (*A*_600_) was measured to estimate the number of CFU, which was verified after overnight culture on TSA (*S. aureus*) and LB (*P. aeruginosa*) plates, respectively.

### Bacterial growth curve kinetics

Bacterial broth cultures of USA300 LAC::lux or Xen41 were prepared as described above. Overnight cultures were diluted 1:50 in their respective growth media. Next, 50 μl of bacterial cultures was incubated with either 50 μl of vehicle (sterile PBS) or 50 μl of TNFR2 agonist to reach final concentrations of 80, 160, and 320 μg/ml in a total volume of 100 μl. The bacterial growth (OD_600_) was measured at 20-min intervals in triplicate for 10 hours while shaking at 282 cpm (counts per minute) at 37°C in a Gen5 plate reader (BioTek).

### Mice

Six- to 10-week-old female WT, TNF^−/−^, TNFR1^−/−^, TNFR2^−/−^, and PAD4^−/−^ mice on a C57BL/6 background were obtained from Jackson Laboratories (Bar Harbor, ME) and used in all experiments for the mouse models of *S. aureus* intradermal infection. All mouse strains were bred and maintained under the same specific pathogen–free conditions, with air-isolated cages at an American Association for the Accreditation of Laboratory Animal Care–accredited animal facility at Johns Hopkins University and handled according to procedures described in the Guide for the Care and Use of Laboratory Animals as well as Johns Hopkins University’s policies and procedures as outlined in the Johns Hopkins University Animal Care and Use Training Manual.

### In vivo BLI

In vivo BLI was performed to approximate the in vivo bacterial burden, as previously described (*16*, *84*). Briefly, anesthetized mice (2% isoflurane) were imaged using a Lumina III IVIS (PerkinElmer), and total flux (photons/s) was measured within a 1 × 10^3^ pixel square region of interest using Living Image software (PerkinElmer; limit of detection: 2 × 10^4^ photons/s) with USA300 LAC::lux or Xen41 bioluminescent bacterial strains. The bioluminescent signals detected from infected skin using USA300 LAC::lux closely approximated the actual bacterial burden measured by ex vivo CFU counting, as previously described (*R*^2^ = 0.9996) (*17*).

### Mouse serum and skin protein isolation for enzyme-linked immunosorbent assay

Mouse blood was collected on the indicated days through retro-orbital bleeding using heparinized microhematocrit capillary tubes (Thermo Fisher Scientific of anesthetized mice (2% isoflurane). Sera were collected by centrifuging the clotted blood for 10 min at 1500*g* and 4°*C. *Sera were then frozen at −80°C until needed. Mouse skin protein was collected by homogenizing a 10 mm skin punch biopsy in RIPA Lysis and Extraction Buffer (Thermo Fisher Scientific) supplemented with Halt Protease and Phosphatase Inhibitor Cocktail (Thermo Fisher Scientific). Skin protein was isolated by collecting the supernatant after centrifuging the skin homogenate (1500*g*) for 5 min at 4°C and frozen at −80°C until needed. Mouse serum and skin TNF levels were quantified using a mouse TNF-alpha Quantikine ELISA Kit, according to the manufacturer’s instructions (R&D Systems).

### Ex vivo kidney CFU enumeration

Kidney bacterial CFU was obtained by isolating both kidneys of intradermally infected mice and homogenized with a Pro200 Series homogenizer (Pro Scientific) in sterile PBS. Ex vivo CFUs of USA300 LAC::lux were counted after plating serially diluted kidney homogenates overnight on TSA plates.

### Histologic analysis of mouse skin

Punch biopsy specimens (10 mm) were placed in 10% formalin-fixed and paraffin-embedded (FFPE). Skin cross sections (4 μm) were prepared onto Superfrost Plus microscope slides (CardinalHealth) and stained with H&E or Gram stain by the Johns Hopkins Reference Histology Laboratory, according to clinical specimen guidelines. Skin sections were digitally scanned using a NanoZoomer XR (Hamamatsu, Japan), and images were exported at ×12.5 magnification with a millimeter-scale bar as a reference. Neutrophilic abscess area and bacterial band width were measured by bright-field microscopy images taken at ×12.5 magnification (Leica, DFC495) with a millimeter-scale bar as a reference using ImageJ software (NIH).

### Flow cytometry

Ten-millimeter punch biopsies of mouse dorsal skin were excised, minced, and placed in 3 ml of RPMI containing DNase I (100 μg/ml; DN25, Sigma-Aldrich, St. Louis, MO) and 1.67 Wunsch units/ml Liberase TL (Roche, Basel, Switzerland). Single-cell suspensions were acquired by digesting the skin for 1 hour at 37°C shaken at 70 rpm and filtering the digested samples through a 40-μm filter using a 3-ml syringe plunger. The cells were then washed once with RPMI and resuspended with PBS. The single-cell suspension was incubated with Viobility Fixable Dye (Miltenyi) to assess cell viability and TruStain FcX (BioLegend) to block Fc receptor binding. The following antibodies were resuspended in PBS supplemented with 1% bovine serum albumin (BSA) and 5 mM EDTA (FACS buffer) to detect cell surface markers: VioBlue and APCVio770-anti-CD45 (REA737); PerCPVio700 and APC-anti-CD11b (REA592); phycoerythrin (PE), FITC, and APCVio770-anti-Ly6G (REA526); VioBlue and APCVio770-anti-Ly6C (REA796); PEVio770-anti-F4/80 (REA126); PE-anti-CD120a (TNFR1) (55R-286; BioLegend); PEVio770 and PerCPVio770-anti-CD120b (TNFR2) (REA252) (all from Miltenyi Biotec, otherwise indicated). Intracellular staining was performed by fixing and permeabilizing single-cell suspensions in BD Cytofix/Cytoperm for 20 min at 4°C and then stained with FITC–anti-TNF (REA636, Miltenyi Biotec). Cell acquisition was performed on a MACSQuant flow cytometer (Miltenyi Biotec), and data were analyzed using MACSQuantify software (Miltenyi Biotec) and Cytobank software (Cytobank). Cell types were defined by flow cytometry according to the following gating strategies: monocytes (Ly6C^+^Ly6G^−^), macrophages (F4/80^+^), and neutrophils (Ly6C^int/low^Ly6G^+^) were identified from the CD45^+^CD11b^+^ population from live single cells (fig. S2). Nonhematopoietic cells were identified as the CD45^−^ population from live single cells. TNF-, TNFR1- and TNFR2-expressing cells were first gated on live, single cells followed by surface markers to identify monocytes, macrophages, neutrophils, and nonhematopoietic cells as detailed above (fig. S4).

For identifying suicidal and vital NETs, single-cell suspension was incubated with Viobility Fixable Dye (Miltenyi) to assess cell viability and 5 μM Sytox Orange (Invitrogen) to detect extracellular double-stranded DNA. Then, cells were fixed with BD Cytofix Fixation buffer for 20 min at 4°C and stained with the following primary antibodies in FACS buffer: anti–histone H3 (citrulline R2 + R8 + R17) (H3-Cit) antibody (rabbit polyclonal, Abcam); biotin anti-myeloperoxidase antibody (2D4, Abcam), VioBlue–anti-CD45 (REA737); PerCPVio700–anti-CD11b (REA592); APCVio770–anti-Ly6G (REA526) (all from Miltenyi Biotec, otherwise indicated). Cells were washed and stained with secondary antibodies Donkey anti-Rabbit Alexa Fluor 647 (polyclonal, Abcam) and Alexa Fluor 488 Streptavidin (Biolegend) to detect surface H3-Cit and MPO, respectively. Suicidal and vital NETs were defined by flow cytometry according to the following gating strategies: neutrophils (CD11b^+^Ly6G^+^) were first gated from singlet CD45^+^CD11b^+^ cells. Then, suicidal and vital NETs were differentiated by dead and live neutrophils, respectively, followed by identification as MPO^+^H3-Cit^+^ neutrophils that had extracellular dsDNA (Sytox Orange^+^) (fig. S5).

### Immunofluorescence microscopy

To identify NETs in the skin ex vivo, FFPE from day 3 after infection were deparaffinized with heat-mediated antigen-retrieval in Trilogy buffer (Cell Marque) and blocked for 1 hour at room temperature with PBS supplemented with 0.5% Tween 20 (PBS-T) and 10% fetal bovine serum (FBS). Blocked slides were then incubated overnight at 4°C with rat anti-Ly6G (2.5 μg/ml; 1A8, BD) and rabbit–anti-H3-cit (2 μg/ml; polyclonal, Abcam) diluted in 10% FBS PBS-T buffer. Stained slides were washed three times with PBS-T and incubated first for 1 hour at room temperature with secondary antibodies goat anti-rat immunoglobulin G (IgG) Alexa Fluor 594 (2 μg/ml; Invitrogen) and goat anti-rabbit IgG Alexa Fluor 488 (2 μg/ml; Invitrogen) diluted in 10% FBS PBS-T buffer. After secondary antibody incubation, slides were washed three times with PBS-T, as previously described. Nuclei were stained with 4′,6-diamidino-2-phenylindole (DAPI) (1 μg/ml in PBS) for 5 min, washed twice with PBS, and subsequently mounted in ProLong Diamond Antifade Mountant (Thermo Fisher Scientific).

To determine activated p47_phox_ in the skin ex vivo, FFPE skin sections were deparaffinized and blocked for 1 hour at room temperature with PBS-T supplemented with 1% BSA. Skin sections were then stained with rat anti-Ly6G (2.5 μg/ml; 1A8, BD) and rabbit anti–phos-p47 (pSer345) (10 μg/ml; polyclonal, Sigma-Aldrich) diluted in 0.5% BSA PBS-T overnight at 4°C. Stained slides were washed three times with PBS-T and incubated first for 1 hour at room temperature with secondary antibodies goat anti-rat IgG Alexa Fluor 594 (2 μg/ml; Invitrogen) and goat anti-rabbit IgG Alexa Fluor 488 (2 μg/ml; Invitrogen) diluted in 0.5% BSA PBS-T buffer. Nuclei were stained with DAPI (1 μg/ml in PBS) for 5 min, washed twice with PBS, and subsequently mounted in ProLong Diamond Antifade Mountant (Thermo Fisher Scientific).

### Immunofluorescence quantification

For H3-Cit quantification, at least three images of the neutrophilic abscess (defined by positive Ly6G stain) for each skin section were taken at ×400 magnification using a fluorescence microscope (DFC365FX, Leica Microsystems). The fluorescence intensity of H3-cit stain was quantified by averaging the integrated density of whole images using ImageJ software (NIH).

For activated p47_phox_ quantification, at least eight images of the neutrophilic abscess for each skin section were taken at ×400 magnification using a fluorescence microscope (DFC365FX, Leica Microsystems). For each image, the number of punctate stains was quantified by normalizing the total count of punctate stains to the area of whole image using ImageJ software (NIH). Then, the total number of activated p47_phox_ per skin section was determined by averaging the number of punctate stains from all images acquired for each skin section.

### BM reconstitution generation

WT or TNF^−/−^ mice were given a lethal dose of 8-Gy irradiation with the CIXD Biological Irradiator (XStrahl), after which 1 × 10^7^ BM cells were administered via retro-orbital vein injection within 3 hours following irradiation. For 3 weeks after irradiation, mice were given drinking water supplemented with Elanco Baytril 100 (0.5 mg/ml; Elanco Animal Health). After 7 to 8 weeks following irradiation, BM reconstitution was confirmed by flow cytometric analysis for TNF-producing cells in the blood.

### Neutrophil isolation and adoptive transfer to mice

To isolate neutrophils, a single-cell suspension of BM cells was obtained from the femurs and tibias of 8- to 10-week-old WT, TNFR1^−/−^, or TNFR2^−/−^ mice by flushing with ≥5 ml of sterile 4°C RPMI media using a 25G needle syringe. BM cells were strained over a 40-μm filter, counted, and resuspended in MACS Separation Buffer [degassed PBS containing BSA (0.5%) and EDTA (2 mM)] (Miltenyi Biotec) to a final concentration of 2.5 × 10^8^ cells/ml. Neutrophils were then purified using a Neutrophil Isolation Kit for mice, according to the manufacturer’s instructions (Miltenyi Biotec). After purification, the cells were > 94% viable and >92% CD45^+^CD11b^+^Ly6G^+^Ly6C^−^ neutrophils (fig. S2). For adoptive transfer experiments, 5.0 × 10^6^ purified neutrophils resuspended in sterile 4°C PBS were injected intravenously via the retro-orbital vein 2 hours before *S. aureus* skin infection.

### In vitro neutrophil ROS quantification

Neutrophils were isolated from BM cells as previously described (refer to the “Neutrophil isolation and adoptive transfer to mice” section). Isolated neutrophils were resuspended to a final concentration of 2.0 × 10^6^ cells/ml in RPMI supplemented with 10% FBS for ROS detection. Cells were seeded into 96-well U-bottom plates at 2.0 × 10^5^ cells in 100 μl per well.

*S. aureus* USA300 LAC::lux bacteria was prepared as previously described (refer to the “Bacterial preparation” section). Multiplicity of infection (MOI) of 100 (2.0 × 10^7^ CFU/ml) were prepared in RPMI supplemented with 10% FBS. Triplicate wells of neutrophils were cultured for 2 hours at 37 ° C with the following reagents: RPMI supplemented with 10% FBS, PMA at 100 ng/ml, and *S. aureus* MOI 100. After stimulation, cells were stained with 100 μl of 20 μM DCFDA dye (Abcam) in RPMI supplemented with 10% FBS for 30 min at 37 ° C. Subsequently, cells were incubated with Viobility Fixable Dye (Miltenyi) to assess cell viability. After staining, cells were washed once and resuspended in 200 μl of FACS buffer for acquisition on the flow cytometer.

### In vitro neutrophil NET quantification

For NET detection, neutrophils were resuspended to a final concentration of 2.0 × 10^6^ cells/ml in R1 medium (RPMI supplemented with 1× minimum essential medium, 1% penicillin/streptomycin, and 1% FBS). Cells were seeded into black 96-well flat-bottom plates at 2.0 × 10^5^ cells in 100 μl per well.

*S. aureus* USA300 LAC::lux bacteria was prepared as previously described (refer to the “Bacterial preparation” section). MOI of 100 (2.0 × 10^7^ CFU/ml) were prepared in R1 medium. Triplicate wells of neutrophils were cultured for 2 hours with the following reagents: R1 medium, PMA (100 ng/ml), *S. aureus* MOI 100. After 2 hours of stimulation, cells were stained with 50 μl of 5 μM SYTOX Green (Thermo Fisher Scientific) in R1 medium for 5 min at room temperature. Cells were then incubated with Viobility Fixable Dye (Miltenyi) to assess cell viability. After staining, cells were washed once and resuspended in 200 μl of FACS buffer for acquisition on the flow cytometer.

### TNFR2 agonist and administration

NewSTAR2 (TNFR2 agonist) was generated as previously described (*45*). Briefly, the TNFR2 agonist NewSTAR2 comprises two single-chain encoded TNFR2-specific murine TNF mutant trimers [sc(mu)TNF80] fused to the C termini of an IgG1 molecule of irrelevant specificity in mice and the N297A mutation that minimizes interaction with Fcγ-receptors. The purified TNFR2 agonist was lipopolysaccharide-free and in the working concentration of 1.4 to 1.6 mg/ml for in vivo experiments. After 4 hours after infection, mice were injected intraperitoneally with 100 to 115 μl of a single dose of 8 mg/kg TNFR2 agonist or vehicle (sterile PBS).

### Study approval

All animal experiments were approved by the Johns Hopkins University Animal Care and Use Committee.

### Statistical analysis

Data comparison between two groups for longitudinal comparisons was compared using a two-way analysis of variance (ANOVA). Single comparisons were compared by using a nonparametric two-tailed Student’s *t* test or a nonparametric Mann-Whitney test. Data between multiple groups (≥3 groups) were compared using a one-way ANOVA multiple comparisons test with Tukey correction or Kruskal-Wallis test with Dunn’s correction as indicated in figure legends. All statistical analysis was calculated with Prism software (GraphPad 9 Software, La Jolla, California). Data are presented as means ± SEM. *P* < 0.05 was considered statistically significant.
