# Supplementary material for: Neutrophil-intrinsic TNF receptor signaling orchestrates host defense against Staphylococcus aureus
Source: Sci Adv. 2023 Jun 16;9(24):eadf8748. doi: 10.1126/sciadv.adf8748 (PMC10275602; doi:10.1126/sciadv.adf8748)
Supplement: Supplementary file 1 — Figs. S1 to S8 [file sciadv.adf8748_sm.pdf]

Science Advances
AAAS

Supplementary Materials for

Neutrophil-intrinsic TNF receptor signaling orchestrates host defense against *Staphylococcus aureus*

Christine Youn *et al.*

Corresponding author: Nathan K. Archer, narcher2@jhmi.edu

Sci. Adv. **9**, eadf8748 (2023)
DOI: 10.1126/sciadv.adf8748

The PDF file includes:

Figs. S1 to S8

Other Supplementary Material for this manuscript includes the following:

Table S1

SUPPLEMENTAL FIGURES

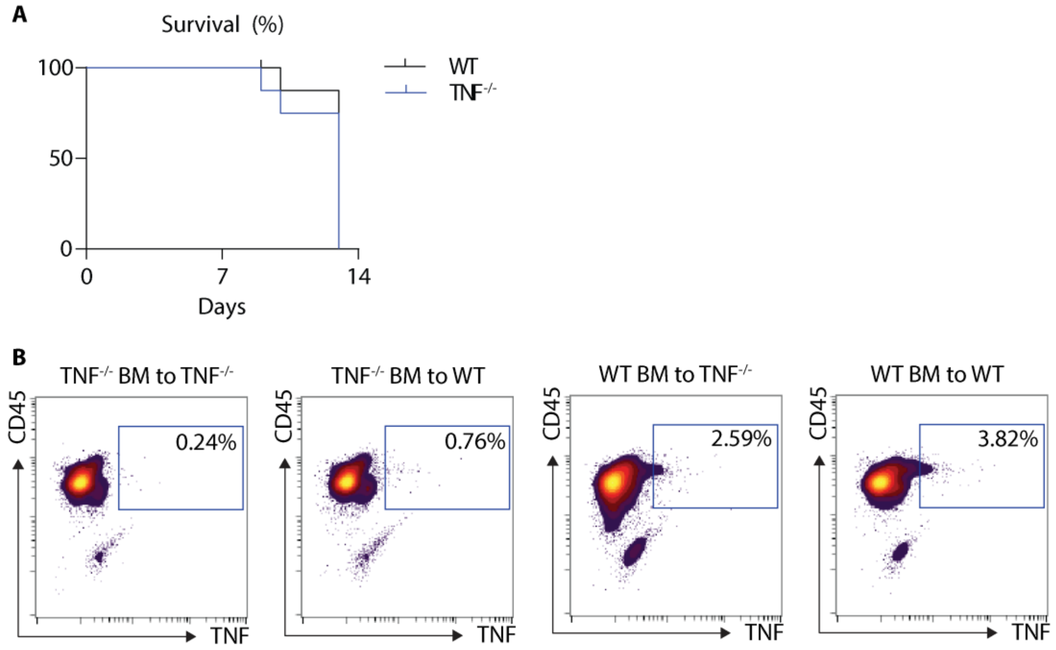

Supplemental Figure 1. Bone marrow reconstitution of WT and TNF$^{-/-}$ mice

(A) Survival rate of mice after irradiation of mice with 8 gy. (B) Representative flow plots for circulating CD45+TNF+ cells (%) 8 weeks after bone marrow reconstitution.

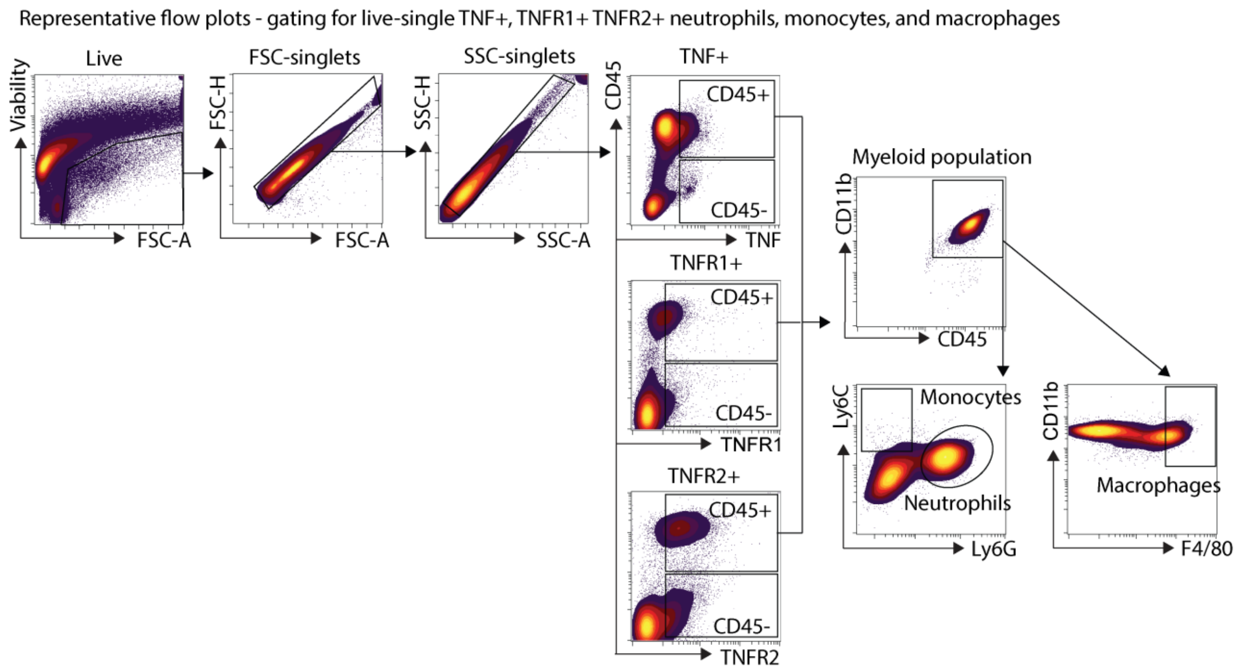

Supplemental Figure 2. Gating strategy for TNF+, TNFR1+, and TNFR2+ cells in the skin

Single cells were isolated from naïve or infected skin at different time points of infection with *S. aureus* and stained with flow cytometric markers for analysis. Representative flow plots for TNF+, TNFR1+, and TNFR2+ monocytes, neutrophils, and macrophages day 3 infected WT skin.

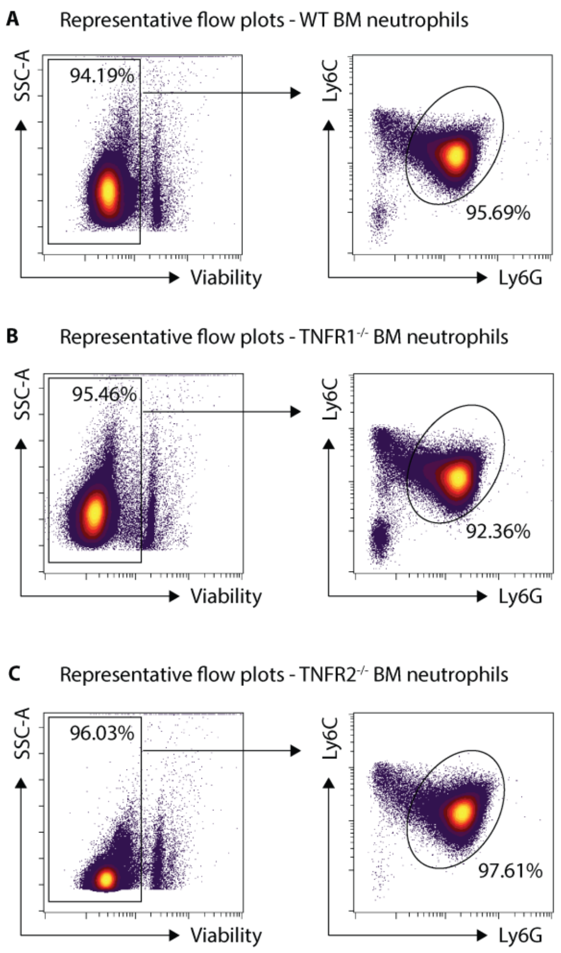

Supplemental Figure 3. Neutrophil adoptive transfer into WT, TNFR1$^{-/-}$, and TNFR2$^{-/-}$ mice

Neutrophils were isolated from 8-10 week old WT, TNFR1$^{-/-}$, and TNFR2$^{-/-}$ mice. Representative flow plots of isolated neutrophils from WT (A), TNFR$^{-/-}$ (B), and TNFR2$^{-/-}$ (C) for viability and purity before transfer into recipient mice.

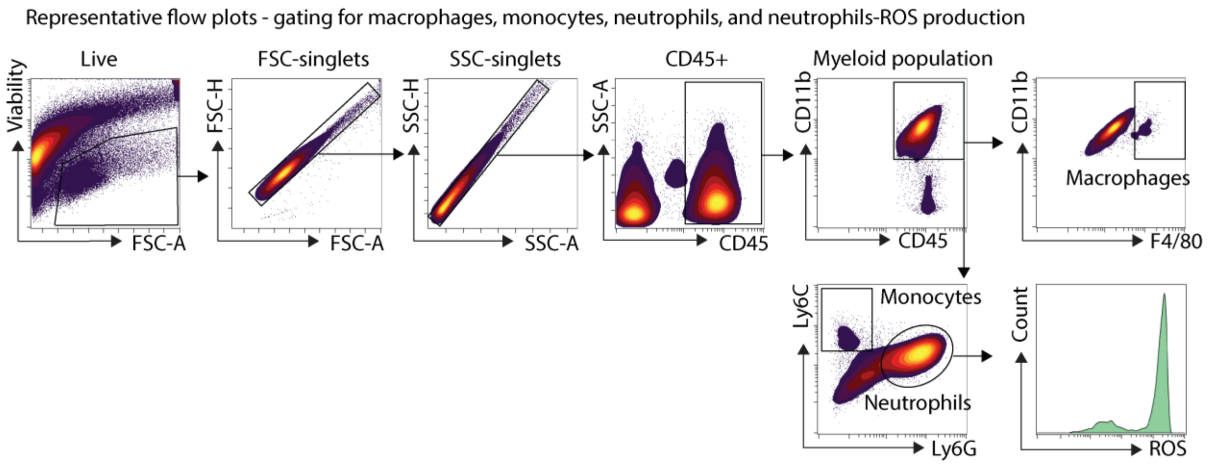

Supplemental Figure 4. Gating strategy for myeloid cell populations and ROS+ neutrophils in the skin

Single cells were isolated from naïve or infected skin at different time points of infection with *S. aureus* and stained with flow cytometric markers for analysis. Representative flow plots showing the gating strategy for monocytes, neutrophils, and macrophages and neutrophil-derived ROS from day 3 infected WT skin.

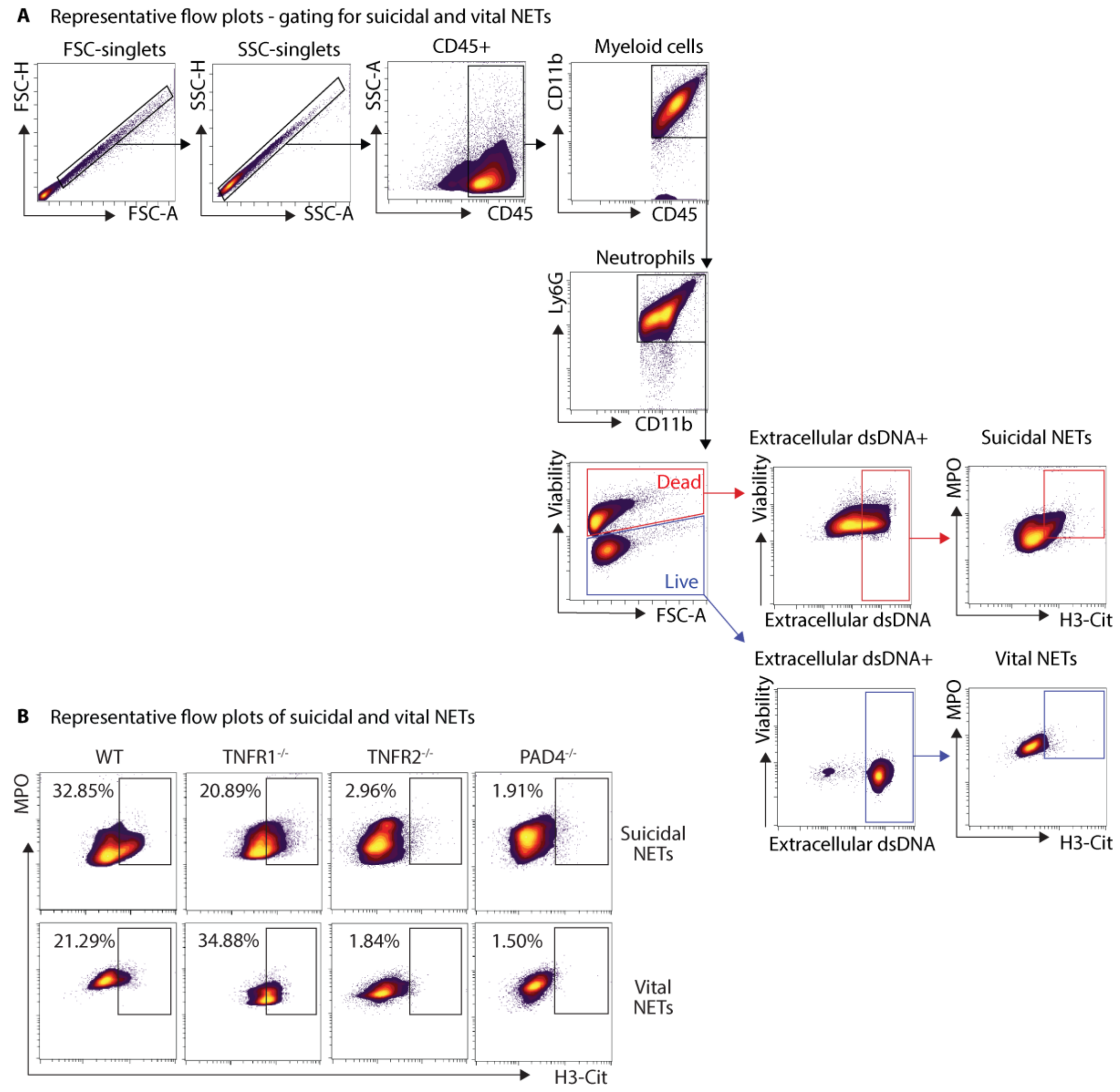

Supplemental Figure 5. Gating strategy for suicidal and vital NETs

(A) Representative flow plots showing the gating strategy for suicidal and vital NETs. (B) Representative flow plots of suicidal and vital NETs from WT, TNF$^{-/-}$, TNFR1$^{-/-}$, TNFR2$^{-/-}$, and PAD4$^{-/-}$ infected day 3 skin.

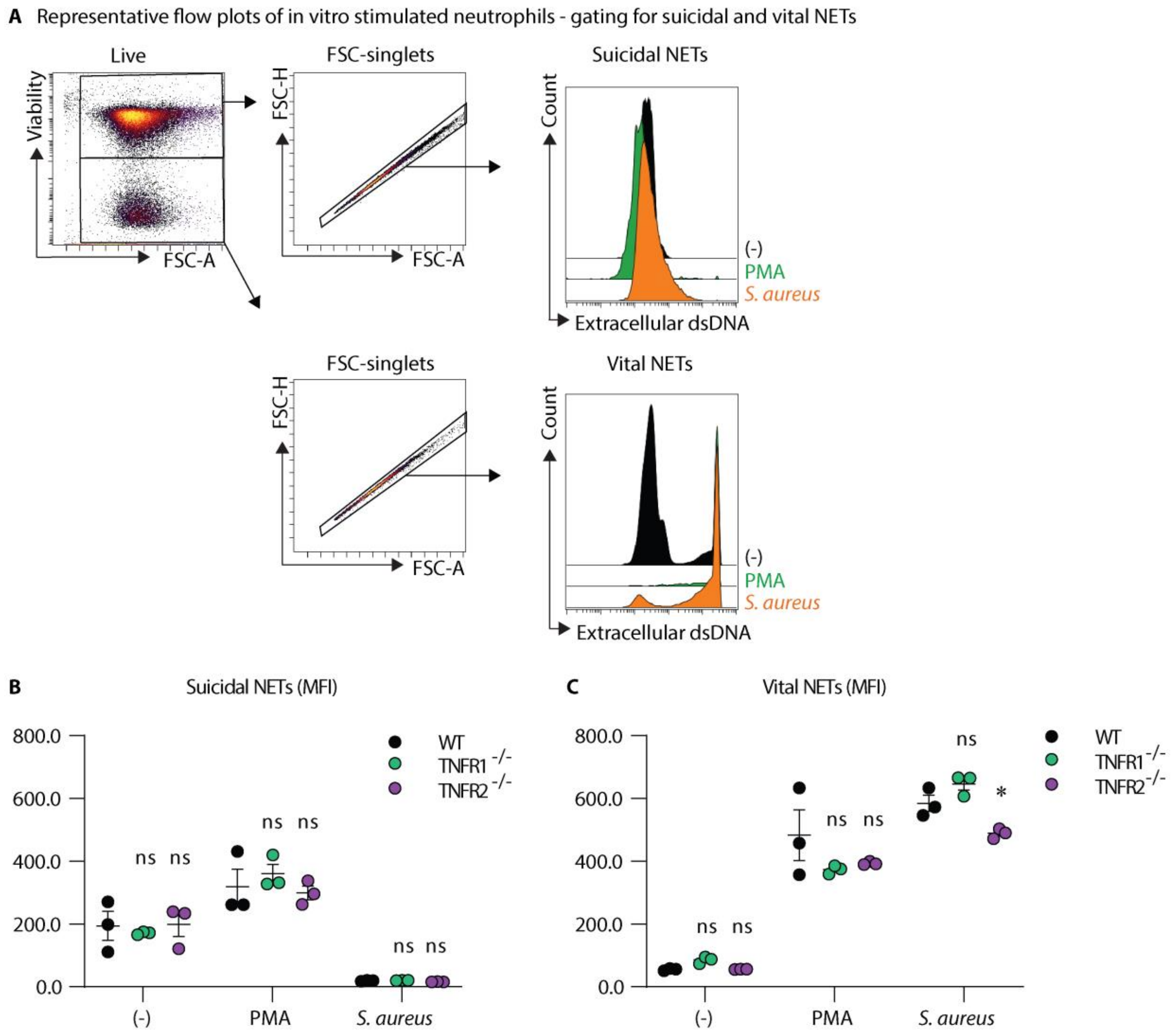

Supplementary Figure 6. TNFR2 is critical for the induction of NETs upon in vitro exposure to *S. aureus*

After 2 hours of incubation with R1 medium (-), PMA, live *S. aureus* at MOI 100, neutrophils were stained with Sytox Green and analyzed by flow cytometry analysis. (A) Representative flow plots showing the gating strategy for suicidal and vital NETs. (B and C) Mean of suicidal NETs (B) and vital NETs (C) mean fluorescence intensity (MFI) ± SEM. **P* < 0.05, as

calculated by Dunnett's one-way ANOVA multiple comparisons test (B and C). ns, not significant. Results are representative of two independent experiments.

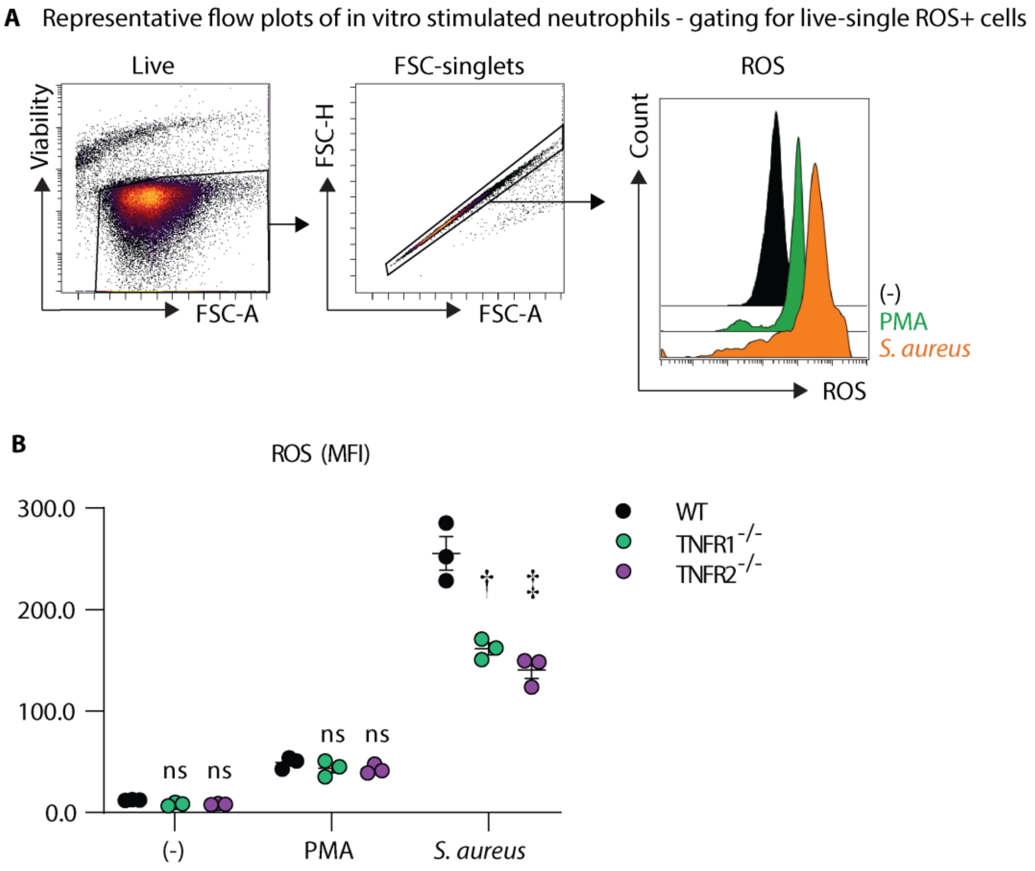

<u>Supplementary Figure 7. TNFR1 and TNFR2 are critical for neutrophil production of ROS upon in vitro stimulation with *S. aureus*</u>

<u>After 2 hours of incubation with RPMI supplemented with 10% FBS (-), PMA, live *S. aureus* at MOI 100, neutrophils were stained with DCFDA dye and analyzed by flow cytometry analysis. (A) Representative flow plots showing the gating strategy for ROS production. (B) Mean of ROS mean fluorescence intensity (MFI) ± SEM. *$P < 0.05$, †$P < 0.01$, ‡$P < 0.001$, as calculated by Dunnett's one-way ANOVA multiple comparisons test (B). ns, not significant. Results are representative of two independent experiments.</u>

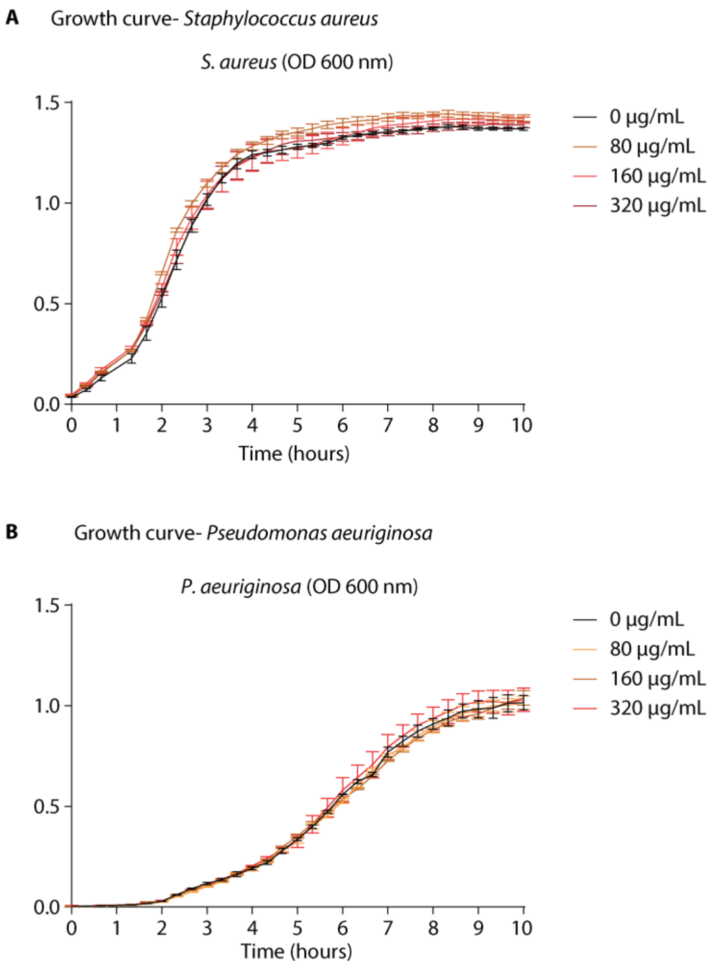

<u>Supplementary Figure 8</u>. The TNFR2 agonist does not have direct antibacterial activity against *S. aureus* and *P. aeruginosa*.

Bacterial broth cultures were incubated with vehicle (0 µg/mL) or various concentrations of TNFR2 agonist (80 µg/mL, 160 µg/mL, and 320 µg/mL). The bacterial growth (OD600) was measured in triplicate (n=3) for 10 hours with measurements recorded at 20-minute intervals. Bacterial growth (OD600) in *S. aureus* (A) and *P. aeruginosa* (B).
